# Refined Regularity of the Blow-Up Set Linked to Refined Asymptotic Behavior for the Semilinear Heat Equation

**DOI:** 10.1515/ans-2016-6005

**Published:** 2017-02-01

**Authors:** Tej-Eddine Ghoul, Van Tien Nguyen, Hatem Zaag

**Affiliations:** ^1^New York University in Abu Dhabi, P.O. Box 129188, Abu Dhabi, United Arab Emirates; ^2^LAGA, CNRS (UMR 7539), Université Paris 13, Sorbonne Paris Cité, 93430 Villetaneuse, France

**Keywords:** Blow-Up Solution, Blow-Up Set, Blow-Up Profile, Regularity, Semilinear Heat Equation, : 35K50, 35B40, : 35K55, 35K57

## Abstract

We consider u⁢(x,t), a solution of $\partial_{t}u=\Delta \,u+{\left|u\right|}^{p-1}\,u$ which blows up at some time T>0, where $u:{\mathbb{R}}^{N}\times \left[0,T\right)\to \mathbb{R}$, p>1 and (N-2)⁢p<N+2. Define $S\subset {\mathbb{R}}^{N}$ to be the blow-up set of *u*, that is, the set of all blow-up points. Under suitable non-degeneracy conditions, we show that if *S* contains an (N-ℓ)-dimensional continuum for some ℓ∈{1,…,N-1}, then *S* is in fact a ${𝒞}^{2}$ manifold. The crucial step is to make a refined study of the asymptotic behavior of *u* near blow-up. In order to make such a refined study, we have to abandon the explicit profile function as a first-order approximation and take a non-explicit function as a first-order description of the singular behavior. This way we escape logarithmic scales of the variable (T-t) and reach significant small terms in the polynomial order ${\left(T-t\right)}^{\mu }$ for some μ>0. Knowing the refined asymptotic behavior yields geometric constraints of the blow-up set, leading to more regularity on *S*.

## 1 Introduction

We are interested in the following semilinear heat equation:

(1.1) $$\left\{ \begin{array}{cc} \partial_{t}u & =\Delta \,u+{\left|u\right|}^{p-1}\,u,\\ u\,\left(0\right) & =u_{0}\in L^{\infty }\,\left({\mathbb{R}}^{N}\right), \end{array}\right.$$

where $u\,\left(t\right):x\in {\mathbb{R}}^{N}\to u\,\left(x,t\right)\in \mathbb{R}$, Δ denotes the Laplacian in ${\mathbb{R}}^{N}$, and p>1 or $1<p<\frac{N+2}{N-2}$ if N≥3. It is well known that for each initial data $u_{0}$ the Cauchy problem (1.1) has a unique solution $u\in 𝒞\,\left(\left[0,T\right),L^{\infty }\,\left({\mathbb{R}}^{N}\right)\right)$ for some 0<T≤+∞, and that either T=+∞ or

$${T\,<+\infty \;\text{and}\;\lim_{t\to T}∥\,u\,\left(t\right)∥}_{L^{\infty }}=+\infty .$$

In the latter case we say that the solution blows up in finite time, and *T* is called the blow-up time. In such a blow-up case, a point $\hat{a}\in {\mathbb{R}}^{N}$ is called a blow-up point if u⁢(x,t) is not locally bounded in some neighborhood of $\left(\hat{a},T\right)$, this means that there exists $\left(x_{n},t_{n}\right)\to \left(\hat{a},T\right)$ such that $\left|u\,\left(x_{n},t_{n}\right)\right|\to +\infty$ when n→+∞. We denote by *S* the blow-up set, that is, the set of all blow-up points of *u*.

Given $\hat{a}\in S$, we know from Velázquez [15] (see also Filippas and Kohn [5], Filippas and Liu [6], Herrero and Velázquez [9], Merle and Zaag [12]) that up to replacing *u* by -u, one of following two cases occurs:

*Case 1 (non-degenerate rate of blow-up).* For all $K_{0}>0$, there is an orthonormal (N×N)-matrix $Q_{\hat{a}}$ and $\ell_{\hat{a}}\in \left\{1,\ldots ,N\right\}$ such that

(1.2) $$\underset{\left|\xi \right|\leq K_{0}}{\sup}\left|{\left(T-t\right)}^{\frac{1}{p-1}}\,u\,\left(\hat{a}+Q_{\hat{a}}\,\xi \,\sqrt{\left(T-t\right)\,\left|\log\left(T-t\right)\right|},t\right)-f_{\ell_{\hat{a}}}\,\left(\xi \right)\right|\to 0\;\text{as }\,t\to T,$$

where

(1.3) $$f_{\ell_{\hat{a}}}\,\left(\xi \right)={\left(p-1+\frac{{\left(p-1\right)}^{2}}{4\,p}\,\sum_{i=1}^{\ell_{\hat{a}}}\xi_{i}^{2}\right)}^{-\frac{1}{p-1}}.$$

*Case 2 (degenerate rate of blow-up).* For all $K_{0}\geq 0$, there exists an even integer m≥4 such that

(1.4) $$\underset{\left|\xi \right|\leq K_{0}}{\sup}\left|{\left(T-t\right)}^{\frac{1}{p-1}}\,u\,\left(\hat{a}+\xi \,{\left(T-t\right)}^{\frac{1}{m}},t\right)-{\left(p-1+\sum_{\left|\alpha \right|=m}c_{\alpha }\,\xi^{\alpha }\right)}^{-\frac{1}{p-1}}\right|\to 0\;\text{as }\,t\to T,$$

where $\xi^{\alpha }=\prod_{i=1}^{N}\xi_{i}^{\alpha_{i}}$, $\left|\alpha \right|=\sum_{i=1}^{N}\alpha_{i}$ if $\alpha =\left(\alpha_{1},\ldots ,\alpha_{n}\right)\in {\mathbb{N}}^{N}$ and $\sum_{\left|\alpha \right|=m}c_{\alpha }\,\xi^{\alpha }\geq 0$ for all $\xi \in {\mathbb{R}}^{N}$.

According to Velázquez [15], if case 1 occurs with $\ell_{\hat{a}}=N$ or case 2 occurs with $\sum_{\left|\alpha \right|=m}c_{\alpha }\,\xi^{\alpha }>0$ for all ξ≠0, then $\hat{a}$ is an isolated blow-up point. Herrero and Velázquez [8, 7] prove that the profile (1.3) with $\ell_{\hat{a}}=N$ is generic in the case N=1, and they announced the same for N≥2, but they never published it. Bricmont and Kupiainen [1] and Merle and Zaag [10] show the existence of initial data for (1.1) such that the corresponding solutions blow up in finite time *T* at only one blow-up point $\hat{a}$ and verify the behavior (1.2) with $\ell_{\hat{a}}=N$. The method of [10] also gives the stability of the profile (1.3) ($\ell_{\hat{a}}=N$) with respect to perturbations in the initial data (see also Fermanian Kammerer, Merle and Zaag [3, 4] for other proofs of the stability). Ebde and Zaag [2] and Nguyen and Zaag [13] prove the stability of the profile (1.3) ($\ell_{\hat{a}}=N$) with respect to perturbations in the initial data and also in the nonlinearity, in some class allowing lower order terms in the solution and also in the gradient. All the other asymptotic behaviors are suspected to be unstable.

When

$$\ell_{\hat{a}}\leq N-1$$

in (1.2), we do not know whether $\hat{a}$ is isolated or not, or whether *S* is continuous near $\hat{a}$. In this paper, we assume that $\hat{a}$ is a non-isolated blow-up point and that *S* is continuous locally near $\hat{a}$, in a sense that we will describe precisely later. Our main concern is the regularity of *S* near $\hat{a}$. The first relevant result is due to Velázquez [16] who shows that the Hausdorff measure of *S* is less than or equal to N-1. No further results on the description of *S* were known until the contributions of Zaag [18, 17, 20] (see also [19] for a summarized note). In [18], he proves that if *S* is locally continuous, then *S* is a ${𝒞}^{1}$ manifold. He also obtains the first description of the singularity near $\hat{a}$. More precisely, he shows in [18, Theorems 3 and 4] that for some $t_{0}<T$ and δ>0, for all $K_{0}>0$, $t\in \left[t_{0},T\right)$ and $x\in B\,\left(\hat{a},2\,\delta \right)$ such that $d\,\left(x,S\right)\leq K_{0}\,\sqrt{\left(T-t\right)\,\left|\log\left(T-t\right)\right|}$, one has

(1.5) $$\left|{\left(T-t\right)}^{\frac{1}{p-1}}\,u\,\left(x,t\right)-f_{1}\,\left(\frac{d\,\left(x,S\right)}{\sqrt{\left(T-t\right)\,\left|\log\left(T-t\right)\right|}}\right)\right|\leq C\,\left(K_{0}\right)\,\frac{\log\left|\log\left(T-t\right)\right|}{\left|\log\left(T-t\right)\right|},$$

where $f_{1}$ is defined in (1.3) ($\ell_{\hat{a}}=1$). Moreover, for all $x\in {\mathbb{R}}^{N}\setminus S$, one has $u\,\left(x,t\right)\to u^{*}\,\left(x\right)$ as t→T with

(1.6) $$u^{*}\,\left(x\right)∼U\,\left(d\,\left(x,S\right)\right)={\left(\frac{8\,p}{{\left(p-1\right)}^{2}}\,\frac{\left|\log d\,\left(x,S\right)\right|}{d^{2}\,\left(x,S\right)}\right)}^{\frac{1}{p-1}}\;\text{as }\,d\,\left(x,S\right)\to 0\,\text{ and }\,x\in B\,\left(\hat{a},2\,\delta \right).$$

If

$$\ell_{\hat{a}}=1,$$

Zaag [17] further refines the asymptotic behavior (1.5) and gets error terms of order ${\left(T-t\right)}^{\mu }$ for some μ>0. This way, he obtains more regularity on the blow-up set *S*. The key idea is to replace the explicit profile $f_{1}$ in (1.5) by a non-explicit function, say $\tilde{u}\,\left(x_{1},t\right)$, then go beyond all logarithmic scales through scaling and matching. In fact, for $\tilde{u}\,\left(x_{1},t\right)$, Zaag takes a symmetric, one-dimensional solution of (1.1) that blows up at the same time *T* only at the origin, and behaves like (1.2) with $\ell_{\hat{a}}=1$. More precisely, he abandons the explicit profile function $f_{1}$ in (1.5) and chooses a non-explicit function ${\tilde{u}}_{\sigma }\,\left(d\,\left(x,S\right),t\right)$ as a first-order description of the singular behavior, where ${\tilde{u}}_{\sigma }$ is defined by

(1.7) $${\tilde{u}}_{\sigma }\,\left(x_{1},t\right)=e^{-\frac{\sigma }{p-1}}\,\tilde{u}\,\left(e^{-\frac{\sigma }{2}}\,x_{1},T-e^{-\sigma }\,\left(T-t\right)\right).$$

He shows that for each blow-up point *a* near $\hat{a}$, there is an optimal scaling parameter σ=σ⁢(a) so that the difference ${\left(T-t\right)}^{\frac{1}{p-1}}\,\left(u\,\left(x,t\right)-{\tilde{u}}_{\sigma \,\left(a\right)}\,\left(d\,\left(x,S\right),t\right)\right)$ along the normal direction to *S* at *a* is minimized. Hence, if the function ${\tilde{u}}_{\sigma \,\left(a\right)}\,\left(d\,\left(x,S\right),t\right)$ is chosen as a first-order description for u⁢(x,t) near (a,T), we avoid logarithmic scales. More precisely, for all $t\in \left[t_{0},T\right)$ and $x\in B\,\left(\hat{a},2\,\delta \right)$ such that $d\,\left(x,S\right)\leq K_{0}\,\sqrt{\left(T-t\right)\,\left|\log\left(T-t\right)\right|}$, one has

(1.8) $${\left(T-t\right)}^{\frac{1}{p-1}}\,\left|u\,\left(x,t\right)-{\tilde{u}}_{\sigma \,\left(a\right)}\,\left(d\,\left(x,S\right),t\right)\right|\leq C\,{\left(T-t\right)}^{\mu },$$

for some μ>0. Note that any other value of σ≠σ⁢(a) in (1.8) gives an error of logarithmic order of the variable (T-t) (the same as in (1.5)). Exploiting estimate (1.8) yields geometric constraints on *S* which imply the ${𝒞}^{1,\frac{1}{2}-\eta }$-regularity of *S* for all η>0. A further refinement of (1.8) given in [20] yields better estimates in the expansion of u⁢(x,t) near (a,T). Moreover, some terms following in the expansion of u⁢(x,t) near (a,T) contain geometrical information about *S*, resulting in more regularity of *S*, namely the ${𝒞}^{2}$-regularity.

In this work, we want to know whether the ${𝒞}^{2}$-regularity near $\hat{a}$ proven in [20] for $\ell_{\hat{a}}=1$ would hold in the case where *u* behaves like (1.2) near $\left(\hat{a},T\right)$ with

(1.9) $$\ell_{\hat{a}}\in \left\{2,\ldots ,N-1\right\}.$$

Since Zaag obtains the result in [18, 20] only when $\ell_{\hat{a}}=1$, this corresponds to an (N-1)-dimensional blow-up set (the codimension of the blow-up set is one, according to [18]). In our opinion, in those papers the major obstacle towards the case (1.9) lays in the fact that Zaag could not refine the asymptotic behavior (1.2) with $\ell_{\hat{a}}\in \left\{2,\ldots ,N-1\right\}$ to go beyond all logarithmic scales and get a smaller error term in polynomial orders of the variable (T-t). It happens that a similar difficulty was already encountered by Fermanian Kammerer and Zaag in [4], when they wanted to find a sharp profile in the case (1.2) with $\ell_{\hat{a}}=N$, which corresponds to an isolated blow-up point, as we have pointed out right after estimate (1.4). Such a sharp profile could be obtained in [4] only when N=1 (which corresponds also to $\ell_{\hat{a}}=1$): unsurprisingly it was ${\tilde{u}}_{\sigma }\,\left(x_{1},t\right)$, the dilated version of $\tilde{u}\,\left(x_{1},t\right)$, the one-dimensional blow-up solution mentioned between estimates (1.6) and (1.7). As a matter of fact, the use of $\tilde{u}\,\left(x_{1},t\right)$ was first used in [4] for the isolated blow-up point in one space dimension (N=1 and $\ell_{\hat{a}}=1$), then later in higher dimensions with an (N-1)-dimensional blow-up surface (N≥2 and still $\ell_{\hat{a}}=1$) in [17].

The interest of $\tilde{u}\,\left(x_{1},t\right)$ is that it provides a one-parameter family of blow-up solutions, thanks to the scaling parameter in (1.7), which enables us to get the sharp profile by suitably choosing the parameter.

Handling the case $\ell_{\hat{a}}\geq 2$ remained open, both for the case of an isolated point ($\ell_{\hat{a}}=N\geq 2$) and a non-isolated blow-up point ($\ell_{\hat{a}}=2,\ldots ,N-1$). From the refinement of the expansion around the explicit profile in $f_{\ell_{\hat{a}}}$ in (1.2), it appeared that one needs a $\ell_{\hat{a}}\,\left(\ell_{\hat{a}}+1\right)/2$-parameter family of blow-up solutions obeying (1.2).

Such a family was constructed by Nguyen and Zaag in [14], and successfully used to derive a sharp profile in the case of an isolated blow-up point ($\ell_{\hat{a}}=N\geq 2$), by fine-tuning the $\ell_{\hat{a}}\,\left(\ell_{\hat{a}}+1\right)/2=N\,\left(N+1\right)/2$ parameters.

In this paper, we aim at using that family to handle the case of a non-isolated blow-up point (N≥2 and $\ell_{\hat{a}}=2,\ldots ,N-1$), in order to generalize the results of Zaag in [18, 17, 20], proving in particular the $C^{2}$-regularity of the blow-up set, under the hypothesis that it is merely continuous.

The main result in this paper is the following.

Theorem 1.1 ($C^{2}$-Regularity of the Blow-Up Set Assuming $C^{1}$-Regularity)

*Take N≥2 and ℓ∈{1,…,N-1}. Consider *u*, a solution of (1.1) that blows up in finite time *T* on a set *S*. Take $\hat{a}\in S$ where *u* behaves locally as stated in (1.2) with $\ell_{\hat{a}}=\ell$. If *S* is locally a $C^{1}$ manifold of dimension N-ℓ, then it is locally $C^{2}$.*

Remark 1.2 Theorem 1.1 was already proved by Zaag [20] only when ℓ=1. Thus, the novelty of our contribution lays in the case ℓ∈{2,…,N-1} and N≥3.

Under the hypotheses of Theorem 1.1, Zaag [18] already proved that *S* is a ${𝒞}^{1}$ manifold near $\hat{a}$, assuming that *S* is continuous. Therefore, Theorem 1.1 can be restated under a weaker assumption. Before stating this stronger version, let us first clearly describe our hypotheses and introduce some terminology borrowed from [18] (see also [17, 20]). According to Velázquez [15, Theorem 2], we know that for all ϵ>0, there is δ⁢(ϵ)>0 such that

$$S\cap B\,\left(\hat{a},2\,\delta \right)\subset \Omega_{\hat{a},\epsilon }\equiv \left\{x\in {\mathbb{R}}^{N}|\left|P_{\hat{a}}\,\left(x-\hat{a}\right)\right|\geq \left(1-\epsilon \right)\,\left|x-\hat{a}\right|\right\},$$

where $P_{\hat{a}}$ is the orthogonal projection over $\pi_{\hat{a}}$, where

$$\pi_{\hat{a}}=\hat{a}+\mathrm{span}\left\{Q_{\hat{a}}^{T}\,e_{\ell_{\hat{a}}+1},\ldots ,Q_{\hat{a}}^{T}\,e_{N}\right\}$$

is the so-called “weak” tangent plane to *S* at $\hat{a}$. Roughly speaking, $\Omega_{\hat{a},\epsilon }$ is a cone with vertex $\hat{a}$ and shrinks to $\pi_{\hat{a}}$ as ϵ→0. In some “weak” sense, *S* is $\left(N-\ell_{\hat{a}}\right)$-dimensional. In fact, here comes our second hypothesis: we assume there is $\Gamma \in 𝒞\,\left({\left(-1,1\right)}^{N-\ell_{\hat{a}}},{\mathbb{R}}^{N}\right)$ such that $\Gamma \,\left(0\right)=\hat{a}$ and Im⁡Γ⊂S, where Im⁡Γ is at least $\left(N-\ell_{\hat{a}}\right)$-dimensional, in the sense that

(1.10) for all b∈Im⁡Γ, there are (N-ℓa^) independent vectors v1,…,vN-ℓa^ in ℝN andfunctions Γ1,…,ΓN-ℓa^ in 𝒞1⁢([0,1],S) such that Γi⁢(0)=b and Γi′⁢(0)=vi.

Hypothesis (1.10) means that *b* is actually non-isolated in $\left(N-\ell_{\hat{a}}\right)$ independent directions. We assume in addition that $\hat{a}$ is not an endpoint in Im⁡Γ in the sense that

(1.11) $$\begin{array}{c} \text{for all }\epsilon >0\text{, the projection of }\Gamma \,\left({\left(-\epsilon ,\epsilon \right)}^{N-\ell_{\hat{a}}}\right)\text{ on the “weak” tangent plane }\pi_{\hat{a}}\\ \text{at }\hat{a}\text{ contains an open ball centered at }\hat{a}\text{.} \end{array}$$

This is the stronger version of our result:

Theorem 1.1${}^{\prime}$ *Take N≥2 and ℓ∈{1,…,N-1}. Consider *u*, a solution of (1.1) that blows up in finite time *T* on a set *S*. Take $\hat{a}\in S$ where *u* behaves locally as stated in (1.2) with $\ell_{\hat{a}}=\ell$. Consider $\Gamma \in C\,\left({\left(-1,1\right)}^{N-\ell },R^{N}\right)$ such that a^=Γ⁢(0)∈Im⁡Γ⊂S and Im⁡Γ is at least (N-ℓ)-dimensional (in the sense of (1.10)). If $\hat{a}$ is not an endpoint (in the sense of (1.11)), then there are δ>0, $\delta_{1}>0$ and $\gamma \in C^{2}\,\left({\left(-\delta_{1},\delta_{1}\right)}^{N-\ell },R^{\ell }\right)$ such that*

Sδ=S∩B⁢(a^,2⁢δ)=graph⁡(γ)∩B⁢(a^,2⁢δ)=Im⁡Γ∩B⁢(a^,2⁢δ),

*and the blow-up set *S* is a $C^{2}$-hypersurface locally near $\hat{a}$.*

Let us now briefly give the main ideas of the proof of Theorem 1.1. The proof is based on techniques developed by Zaag in [17, 20] for the case when the solution of equation (1.1) behaves like (1.2) with ℓ=1. As in [17, 20], the proof relies on two arguments:

• The derivation of a sharp blow-up profile of u⁢(x,t) near the singularity, in the sense that the difference between the solution u⁢(x,t) and this sharp profile goes beyond all logarithmic scales of the variables (T-t). This is possible thanks to the recent result in [14].
• The derivation of a refined asymptotic profile of u⁢(x,t) near the singularity linked to geometric constraints on the blow-up set. In fact, we derive an asymptotic profile for u⁢(x,t) in every ball $B\,\left(a,K_{0}\,\sqrt{T-t}\right)$ for some $K_{0}>0$ and a blow-up point *a* close to $\hat{a}$. Moreover, this profile is continuous in *a* and the speed of convergence of *u* to the profile in the ball $B\,\left(a,K_{0}\,\sqrt{T-t}\right)$ is uniform with respect to *a*. If *a* and *b* are in *S* and $0<\left|a-b\right|\leq K_{0}\,\sqrt{T-t}$, then the balls $B\,\left(a,K_{0}\,\sqrt{T-t}\right)$ and $B\,\left(b,K_{0}\,\sqrt{T-t}\right)$ intersect each other, leading to different profiles for u⁢(x,t) in the intersection. However, these profiles have to coincide, up to the error terms. This creates a geometric constraint which gives more regularity for the blow-up set near $\hat{a}$.

Let us explain the difficulty raised in [17, 20] for the case ℓ≥2. Consider $a\in S\cap B\,\left(\hat{a},2\,\delta \right)$ for some δ>0 and introduce the following self-similar variables:

(1.12) $$W_{a}\,\left(y,s\right)={\left(T-t\right)}^{\frac{1}{p-1}}\,u\,\left(x,t\right),y=\frac{x-a}{\sqrt{T-t}},s=-\log\left(T-t\right).$$

Then, we see from (1.1) that for all $\left(y,s\right)\in {\mathbb{R}}^{N}\times \left[-\log T,+\infty \right)$,

(1.13) $$\frac{\partial W_{a}}{\partial s}=\Delta \,W_{a}-\frac{1}{2}\,y\cdot \nabla W_{a}-\frac{W_{a}}{p-1}+{\left|W_{a}\right|}^{p-1}\,W_{a}.$$

Under the hypotheses stated in Theorem 1.1, Zaag proved in [18, Proposition 3.1 and pp. 530–533, Section 6.1] that for all $a\in S_{\delta }\equiv S\cap B\,\left(\hat{a},2\,\delta \right)$ for some δ>0 and s≥-log⁡T, there exists an (N×N) orthogonal matrix $Q_{a}$ such that

(1.14) $${∥W_{a}\,\left(Q_{a}\,y,s\right)-\left\{\kappa +\frac{\kappa }{2\,p\,s}\,\left(\ell -\frac{{\left|\bar{y}\right|}^{2}}{2}\right)\right\}∥}_{L_{\rho }^{2}}\leq C\,\frac{\log s}{s^{2}},$$

where $\kappa ={\left(p-1\right)}^{-\frac{1}{p-1}}$, $\bar{y}=\left(y_{1},\ldots ,y_{\ell_{a}}\right)$, $Q_{a}$ is continuous in terms of *a* such that $\left\{Q_{a}^{T}\,e_{j}|j=\ell +1,\ldots ,N\right\}$ spans the tangent plane $\pi_{a}$ to *S* at *a* and $Q_{a}^{T}\,e_{i}$, i=1,…,ℓ are the normal directions to *S* at *a*, $L_{\rho }^{2}$ is the weighted $L^{2}$ space associated with the weight $\rho =\frac{1}{{\left(4\,\pi \right)}^{N/2}}\,e^{-{\left|y\right|}^{2}/4}$. Note that estimate (1.14) implies (1.5) (see [18, Appendix C]).

When ℓ=1, in order to refine estimate (1.14), Zaag in [17] subtracts from $W_{a}$ a one-dimensional solution with the same profile. Let us do the same when ℓ=2,…,N-1, and explain how Zaag succeeds in handing the case ℓ=1 and gets stuck when ℓ≥2. To this end, we consider $\hat{u}\,\left(\bar{x},t\right)$ with $\bar{x}=\left(x_{1},\ldots ,x_{\ell }\right)$ a radially symmetric solution of (1.1) in ${\mathbb{R}}^{\ell }$ which blows up at time *T* only at the origin with the profile (1.2) with $\ell_{\hat{a}}=\ell$ (see [14, Appendix A.1] for the existence of such a solution). If the ℓ-dimensional solution $\hat{u}$ is considered in ${\mathbb{R}}^{N}$, then it blows up on the (N-ℓ)-dimensional vector space $\left\{\bar{x}=0\right\}$ in ${\mathbb{R}}^{N}$. In particular, if we introduce

(1.15) $$\hat{w}\,\left(\bar{y},s\right)={\left(T-t\right)}^{\frac{1}{p-1}}\,\hat{u}\,\left(\bar{x},t\right),\bar{y}=\frac{\bar{x}}{\sqrt{T-t}},s=-\log\left(T-t\right),$$

then $\hat{w}$ is a radially symmetric solution of (1.13) which satisfies

(1.16) $${∥\hat{w}\,\left(\bar{y},s\right)-\left\{\kappa +\frac{\kappa }{2\,p\,s}\,\left(\ell -\frac{{\left|\bar{y}\right|}^{2}}{2}\right)\right\}∥}_{L_{\rho }^{2}}\leq C\,\frac{\log s}{s^{2}}.$$

Noting that $\hat{u}$ and $\hat{w}$ may be considered as solutions defined for all $y\in {\mathbb{R}}^{N}$ (and independent of $y_{\ell +1},\ldots ,y_{N}$), and given that $\hat{w}\,\left(\bar{y},s\right)$ and $W_{a}\,\left(Q_{a}\,y,s\right)$ have the same behavior up to the first order (see (1.14) and (1.16)), we may try to use $\hat{w}$ as a sharper (though non-explicit) profile for $W_{a}\,\left(Q_{a}\,y,s\right)$. In fact, we have the following classification (see Corollary 2.2 below):

*Case 1.* There is a symmetric, real $\left(\ell_{a}\times \ell_{a}\right)$-matrix ℬ=ℬ⁢(a)≠0 such that

(1.17) $$W_{a}\,\left(Q_{a}\,y,s\right)-\hat{w}\,\left(\bar{y},s\right)=\frac{1}{s^{2}}\,\left(\frac{1}{2}\,{\bar{y}}^{T}\,ℬ\,\bar{y}-\mathrm{tr}\left(ℬ\right)\right)+o\,\left(\frac{1}{s^{2}}\right)\;\text{as }\,s\to +\infty \;\text{in }\,L_{\rho }^{2}.$$

*Case 2.* There is a positive constant $C_{0}$ such that

(1.18) $${∥W_{a}\,\left(Q_{a}\,y,s\right)-\hat{w}\,\left(\bar{y},s\right)∥}_{L_{\rho }^{2}}=𝒪\,\left(e^{-\frac{s}{2}}\,s^{C_{0}}\right)\;\text{as }\,s\to +\infty .$$

If ℓ=1 (ℬ⁢(a)∈ℝ), Zaag in [17] noted the following property:

(1.19) $$\hat{w}\,\left(y_{1},s+\sigma_{0}\right)-\hat{w}\,\left(y_{1},s\right)=\frac{2\,\kappa \,\sigma_{0}}{p\,s^{2}}\,\left(\frac{1}{2}\,y_{1}^{2}-1\right)+o\,\left(\frac{1}{s^{2}}\right)\;\text{in }\,L_{\rho }^{2}.$$

Therefore, choosing $\sigma_{0}\,\left(a\right)$ such that $\frac{2\,\kappa \,\sigma_{0}}{p}=ℬ\,\left(a\right)$, we see from (1.17) and (1.19) that

$$W_{a}\,\left(Q_{a}\,y,s\right)-\hat{w}\,\left(y_{1},s+\sigma_{0}\,\left(a\right)\right)=o\,\left(\frac{1}{s^{2}}\right)\;\text{as }\,s\to +\infty \;\text{in }\,L_{\rho }^{2}.$$

From the classification given in (1.17) and (1.18), only (1.18) holds and

(1.20) $${∥W_{a}\,\left(Q_{a}\,y,s\right)-\hat{w}\,\left(y_{1},s+\sigma_{0}\,\left(a\right)\right)∥}_{L_{\rho }^{2}}=𝒪\,\left(e^{-\frac{s}{2}}\,s^{C_{0}}\right)\;\text{as }\,s\to +\infty .$$

If we return to the original variables u⁢(x,t) and $\hat{u}\,\left(x_{1},t\right)$ through (1.12) and (1.15), then (1.8) follows from the transformation (1.7) together with estimate (1.20) (see [17, Appendix C]). In other words, $\hat{w}\,\left(y_{1},s+\sigma_{0}\,\left(a\right)\right)$ serves as a sharp (though non-explicit) profile for $W_{a}\,\left(Q_{a}\,y,s\right)$ in the sense of (1.20). Using estimate (1.20) together with some geometrical arguments, we are able to prove the ${𝒞}^{1,\frac{1}{2}-\eta }$-regularity of the blow-up set, for any η>0. Then, a further refinement of (1.20) up to order of $e^{-\frac{s}{2}}/s$ together with a geometrical constraint on the blow-up set *S* results in more regularity for *S*, which yields the ${𝒞}^{2}$-regularity.

If ℓ≥2, the matrix ℬ⁢(a) in (1.17) has $\frac{\ell \,\left(\ell +1\right)}{2}$ real parameters. Therefore, applying the trick of [17] (see (1.19) above) only allows us to control one parameter; there remain $\frac{\ell \,\left(\ell +1\right)}{2}-1$ real parameters to be handled. This is the major reason which prevents Zaag in [17, 20] from deriving a similar estimate to (1.20), hence, the refined regularity of the blow-up set. Fortunately, we can overcome this obstacle thanks to a recent result by Nguyen and Zaag (see Proposition 2.4 below) who show in [14] that for any symmetric, real (ℓ×ℓ)-matrix 𝒜, there is a solution $w_{𝒜}$ of equation (1.13) in ${\mathbb{R}}^{\ell }$ such that

(1.21) $$w_{𝒜}\,\left(\bar{y},s\right)-\hat{w}\,\left(\bar{y},s\right)=\frac{1}{s^{2}}\,\left(\frac{1}{2}\,{\bar{y}}^{T}\,𝒜\,\bar{y}-\mathrm{tr}\left(𝒜\right)\right)+o\,\left(\frac{1}{s^{2}}\right)\;\text{as }\,s\to +\infty \;\text{in }\,L_{\rho }^{2}.$$

Hence, choosing 𝒜=ℬ⁢(a), we see from (1.21), (1.17) and (1.18) that

(1.22) $${∥W_{a}\,\left(Q_{a}\,y,s\right)-w_{ℬ\,\left(a\right)}\,\left(\bar{y},s\right)∥}_{L_{\rho }^{2}}\leq C\,e^{-\frac{s}{2}}\,s^{C_{0}}$$

for *s* large enough. Exploiting estimate (1.22) and adapting the arguments given in [17, 20], we are able to prove the ${𝒞}^{2}$-regularity of the blow-up set.

The next result shows how the ${𝒞}^{2}$-regularity is linked to the refined asymptotic behavior of $W_{a}$. More precisely, we link in the following theorem the refinement of the asymptotic behavior of $W_{a}$ to the second fundamental form of the blow-up set at *a*.

Theorem 1.3 **(Refined Asymptotic Behaviors Linked to the Geometrical Description of the Blow-Up Set)**

*Under the hypotheses of Theorem 1.1, there exist ${\tilde{s}}_{0}\geq -\log T$ and δ>0 such that for all $a\in S_{\delta }=S\cap B\,\left(\hat{a},2\,\delta \right)$, there exists a symmetric (ℓ×ℓ) matrix B⁢(a) such that for all $s\geq {\tilde{s}}_{0}$,*

(1.23) $${∥W_{a}\,\left(Q_{a}\,y,s\right)-w_{ℬ\,\left(a\right)}\,\left(\bar{y},s\right)-\frac{\kappa \,e^{-\frac{s}{2}}}{2\,p\,s}\,\sum_{i=1}^{\ell }y_{i}\,\sum_{k,j=\ell +1}^{N}\frac{\Lambda_{k,j}^{\left(i\right)}\,\left(a\right)}{1+\delta_{k,j}}\,\left(y_{k}\,y_{j}-2\,\delta_{k,j}\right)∥}_{L_{\rho }^{2}}\leq C\,\frac{e^{-\frac{s}{2}}}{s^{\frac{3}{2}-\nu }},$$

*for some $\nu \in \left(0,\frac{1}{2}\right)$, where $a\to {\left\{\Lambda_{k,j}^{\left(i\right)}\,\left(a\right)\right\}}_{\ell +1\leq j,k\leq N}$ is a continuous symmetric matrix representing the second fundamental form of the blow-up set at the blow-up point *a* along the unitary normal vector $Q_{a}^{T}\,e_{i}$. Moreover,*

(1.24) $$\Lambda_{k,j}^{\left(i\right)}\,\left(a\right)=\frac{p}{4\,\kappa }\,\lim_{s\to +\infty }s\,e^{\frac{s}{2}}\,\int_{{\mathbb{R}}^{N}}W_{a}\,\left(Q_{a}\,y,s\right)\,y_{i}\,\left(y_{k}\,y_{j}-2\,\delta_{k,j}\right)\,\rho \,\left(y\right)\,𝑑y.$$

In Section 2, we give the main steps of the proofs of Theorems 1.1 and 1.3. We leave all long and technical proofs to Section 3.

## Setting of the Problem and Strategy of the Proof of the ${𝒞}^{2}$-regularity of the Blow-Up Set

2

In this section we give the main steps of the proofs of Theorems 1.1 and 1.3. All long and technical proofs will be left to the next section. We proceed in three parts corresponding to three separate subsections. For the reader’s convenience, we briefly describe these parts as follows:

• Part 1: We derive a sharp blow-up behavior for solutions of equation (1.1) having the profile (1.2) with $\ell_{\hat{a}}\in \left\{1,\ldots ,N-1\right\}$ such that the difference between the solution and this sharp blow-up behavior goes beyond all logarithmic scales of the variable T-t. The main result in this step is stated in Proposition 2.5.
• Part 2: Through the introduction of a local chart, we give a geometrical constraint on the expansion of the solution linked to the asymptotic behavior (see Proposition 2.7). This geometrical constraint is a crucial point which is the bridge between the asymptotic behavior and the regularity of the blow-up set.
• Part 3: Using the sharp blow-up behavior derived in Part 1, we first get the ${𝒞}^{1,\frac{1}{2}-\eta }$-regularity of the blow-up set *S* (see Proposition 2.8), then together with the geometrical constraint, we achieve the ${𝒞}^{1,1-\eta }$-regularity of *S* (see Proposition 2.9). With this better regularity and the geometric constraint, we further refine the asymptotic behavior (see Proposition 2.10) and use again the geometric constraint to get ${𝒞}^{2}$-regularity of *S*, which yields the conclusion of Theorems 1.1 and 1.3.

We remark that Parts 1 and 2 are independent, whereas Part 3 is a combination of the first two. Throughout this paper, we work under the hypotheses of Theorem 1.1. Since *S* is locally near $\hat{a}$ a manifold of dimension N-ℓ, we may assume that there is a ${𝒞}^{1}$ function γ such that

$$S_{\delta }\equiv S\cap B\,\left(\hat{a},2\,\delta \right)=\mathrm{graph}\left(\gamma \right)\cap B\,\left(\hat{a},2\,\delta \right),$$

for some δ>0 and $\gamma \in {𝒞}^{1}\,\left({\left(-\delta_{1},\delta_{1}\right)}^{N-\ell },{\mathbb{R}}^{\ell }\right)$ with $\delta_{1}>0$.

In what follows, ℓ∈{1,…,N-1} is fixed, and for all $z=\left(z_{1},\ldots ,z_{N}\right)\in {\mathbb{R}}^{N}$, we denote by $\bar{z}$ the first ℓ coordinates of *z*, namely $\bar{z}=\left(z_{1},\ldots ,z_{\ell }\right)$, and by $\tilde{z}$ the last (N-ℓ) coordinates of *z*, namely $\tilde{z}=\left(z_{\ell +1},\ldots ,z_{N}\right)$. We usually use indices *i*, *m* for the range 1,…,ℓ and indices *j*, *k*, *n* for the range ℓ+1,…,N.

### Part 1: Blow-Up Behavior Beyond All Logarithmic Scales of (T-t)

2.1

In this subsection, we use the ideas given by Fermanian Kammerer and Zaag [4] together with a recent result by Nguyen and Zaag in [14] in order to derive a sharp (though non-explicit) profile for blow-up solutions of (1.1) in the sense that the first order in the expansion of the solution around this sharp profile goes beyond all logarithmic scales of (T-t) and reaches polynomial scales of (T-t). In fact, we replace the 1-scaling parameter σ in (1.8) by a $\frac{\ell \,\left(\ell +1\right)}{2}$-parameters family, which generates a substitution for ${\tilde{u}}_{\sigma }$ defined in (1.7) and serves as a sharp profile for solutions having the behavior (1.2) with $\ell_{\hat{a}}\in \left\{1,\ldots ,N-1\right\}$. The main result in this part is Proposition 2.5 below.

Consider $a\in S_{\delta }$. If $W_{a}\,\left(y,s\right)$ and $\hat{w}\,\left(\bar{y},s\right)$ are defined as in (1.12) and (1.15), then we know from [18] that

(2.1) $${∥W_{a}\,\left(Q_{a}\,y,s\right)-\left\{\kappa +\frac{\kappa }{2\,p\,s}\,\left(\ell -\frac{{\left|\bar{y}\right|}^{2}}{2}\right)\right\}∥}_{L_{\rho }^{2}}\leq C\,\frac{\log s}{s^{2}}$$

and

(2.2) $${∥\hat{w}\,\left(\bar{y},s\right)-\left\{\kappa +\frac{\kappa }{2\,p\,s}\,\left(\ell -\frac{{\left|\bar{y}\right|}^{2}}{2}\right)\right\}∥}_{L_{\rho }^{2}}\leq C\,\frac{\log s}{s^{2}}.$$

The first step is to classify all possible asymptotic behaviors of $W_{a}\,\left(Q_{a}\,y,s\right)-\hat{w}\,\left(\bar{y},s\right)$ as *s* goes to infinity. To do so, we shall use the following result which is inspired by Fermanian Kammerer and Zaag [4].

Proposition 2.1 **(Classification of the Difference Between Two Solutions of (1.13) Having the Same Profile)**

*Assume that $W_{1}$ and $W_{2}$ are two solutions of (1.13) verifying*

(2.3) $${∥W_{i}\,\left(y,s\right)-\left\{\kappa +\frac{\kappa }{2\,p\,s}\,\left(\ell -\frac{{\left|\bar{y}\right|}^{2}}{2}\right)\right\}∥}_{L_{\rho }^{2}}\leq C\,\frac{\log s}{s^{2}},i=1,2,$$

*where $\bar{y}=\left(y_{1},\ldots ,y_{\ell }\right)$ for some ℓ∈{1,…,N-1}. Then, one of the two following cases occurs:*

•
*Case 1. There is a symmetric, real* (ℓ×ℓ) *-matrix* ℬ≠0 *such that*
(2.4) $$W_{1}\,\left(y,s\right)-W_{2}\,\left(y,s\right)=\frac{1}{s^{2}}\,\left(\frac{1}{2}\,{\bar{y}}^{T}\,ℬ\,\bar{y}-\mathrm{tr}\left(ℬ\right)\right)+o\,\left(\frac{1}{s^{2}}\right)\;\text{as }\,s\to +\infty \;\text{in }\,L_{\rho }^{2}.$$
•
*Case 2.* *There is* $C_{0}>0$ *such that*
$${∥W_{1}\,\left(y,s\right)-W_{2}\,\left(y,s\right)∥}_{L_{\rho }^{2}}=𝒪\,\left(e^{-\frac{s}{2}}\,s^{C_{0}}\right)\;\text{as }\,s\to +\infty .$$

**Proof.**

The proof follows from the strategy given in [4] for the difference of two solutions with the radial profile (ℓ=N). Note that the case when ℓ=1 was treated in [17]. Since some technical details are straightforward, we briefly give the main steps of the proof in Section 3.1 and just emphasize the novelties. ∎

An application of Proposition 2.1 with $W_{1}\,\left(y,s\right)=W_{a}\,\left(Q_{a}\,y,s\right)$ and $W_{2}\,\left(y,s\right)=\hat{w}\,\left(\bar{y},s\right)$ yields the following corollary directly.

Corollary 2.2 *As *s* goes to infinity, one of the two following cases occurs:*

•
*Case 1. There is a symmetric, real* (ℓ×ℓ) *-matrix* ℬ=ℬ⁢(a)≠0 *continuous as a function of* *a* *such that*
(2.5) $$W_{a}\,\left(Q_{a}\,y,s\right)-\hat{w}\,\left(\bar{y},s\right)=\frac{1}{s^{2}}\,\left(\frac{1}{2}\,{\bar{y}}^{T}\,ℬ\,\bar{y}-\mathrm{tr}\left(ℬ\right)\right)+o\,\left(\frac{1}{s^{2}}\right)\;\text{in }\,L_{\rho }^{2}.$$
•
*Case 2. There is* $C_{0}>0$ *such that*
(2.6) $${∥W_{a}\,\left(Q_{a}\,y,s\right)-\hat{w}\,\left(\bar{y},s\right)∥}_{L_{\rho }^{2}}=𝒪\,\left(e^{-\frac{s}{2}}\,s^{C_{0}}\right).$$

Remark 2.3 Note that the continuity of ℬ comes from the continuity of $W_{a}$ with respect to *a*, where $W_{a}$ behaves as in (2.1). In particular, Zaag [18] showed the stability of the blow-up behavior (2.1) with respect to blow-up points (see [18, Proposition 3.1 and Section 6.1]).

In the next step, we recall a recent result by Nguyen and Zaag [14], which gives the construction of solutions for equation (1.13) with some prescribed behavior.

Proposition 2.4 **(Construction of Solutions for (1.13) with Some Prescribed Behavior)**

*Let ℓ∈{1,…,N-1}. For all $A\in M_{\ell }\,\left(R\right)$, where $M_{\ell }\,\left(R\right)$ is the set of all symmetric, real (ℓ×ℓ)-matrices, there exists a solution $w_{A}\,\left(y,s\right)$ of (1.13) defined on $R^{N}\times \left[s_{0}\,\left(A\right),+\infty \right)$ such that*

(2.7) $$w_{𝒜}\,\left(\bar{y},s\right)-\hat{w}\,\left(\bar{y},s\right)=\frac{1}{s^{2}}\,\left(\frac{1}{2}\,{\bar{y}}^{T}\,𝒜\,\bar{y}-\mathrm{tr}\left(𝒜\right)\right)+o\,\left(\frac{1}{s^{2}}\right)\;\text{as }\,s\to +\infty \;\text{in }\,L_{\rho }^{2},$$

*where $\hat{w}$ is the radially symmetric, ℓ-dimensional solution of (1.13) satisfying (2.2).*

**Proof.**

See [14, Theorem 3]. Although that result is stated for the case ℓ=N, we can extend it to the case when ℓ≤N-1 by considering solutions of (1.13) as ℓ-dimensional solutions, those artificially generated by adding irrelevant space variables $\left(y_{\ell +1},\ldots ,y_{N}\right)$ to the domain of definition of the solutions. ∎

The following result is a direct consequence of Corollary 2.2 and Proposition 2.4.

Proposition 2.5 (Sharp (Non-Explicit) Profile for Solutions of (1.1) Having the Behavior (1.2) with ℓ≤N-1)

*There exist $s_{0}>0$ and a continuous matrix $B:S_{\delta }\to M_{\ell }\,\left(R\right)$, such that for all $a\in S_{\delta }$ and $s\geq s_{0}$,*

(2.8) $${∥W_{a}\,\left(Q_{a}\,y,s\right)-w_{ℬ\,\left(a\right)}\,\left(\bar{y},s\right)∥}_{L_{\rho }^{2}}\leq C\,e^{-\frac{s}{2}}\,s^{C_{0}},$$

*where $w_{B}$ is the solution constructed as in Proposition 2.4, $C_{0}>0$ is given in Proposition 2.1. Moreover, we have the following:*

(i) *For all* $s\geq s_{0}+1$ ,
(2.9) $$\underset{\left|y\right|\leq K\,\sqrt{s}}{\sup}\left|W_{a}\,\left(y,s\right)-w_{ℬ\,\left(a\right)}\,\left({\bar{y}}_{a},s\right)\right|\leq C\,\left(K\right)\,e^{-\frac{s}{2}}\,s^{\frac{3}{2}+C_{0}},$$
*where* ${\bar{y}}_{a}=\left(y\cdot Q_{a}\,e_{1},\ldots ,y\cdot Q_{a}\,e_{\ell }\right)$ .
(ii) *For all* $t\in \left[T-e^{-s_{0}-1},T\right)$ ,
(2.10) $$\underset{\left|x-a\right|\leq K\,\sqrt{\left(T-t\right)\,\left|\log\left(T-t\right)\right|}}{\sup}\left|{\left(T-t\right)}^{\frac{1}{p-1}}\,u\,\left(x,t\right)-w_{ℬ\,\left(a\right)}\,\left({\bar{y}}_{a,x},-\log\left(T-t\right)\right)\right|\leq C\,\left(K\right)\,{\left(T-t\right)}^{\frac{1}{2}}\,{\left|\log\left(T-t\right)\right|}^{\frac{3}{2}+C_{0}},$$
*where* ${\bar{y}}_{a,x}=\frac{1}{\sqrt{T-t}}\,\left(\left(x-a\right)\cdot Q_{a}\,e_{1},\ldots ,\left(x-a\right)\cdot Q_{a}\,e_{\ell }\right)$ .

**Proof.**

From (2.5) and (2.7), we have for any symmetric (ℓ×ℓ)-matrix 𝒜,

$$W_{a}\,\left(Q_{a}\,y,s\right)-w_{𝒜}\,\left(\bar{y},s\right)=\frac{1}{s^{2}}\,\left(\frac{1}{2}\,{\bar{y}}^{T}\,\left(ℬ-𝒜\right)\,\bar{y}-\mathrm{tr}\left(ℬ-𝒜\right)\right)+o\,\left(\frac{1}{s^{2}}\right)\;\text{in }\,L_{\rho }^{2}.$$

Choosing 𝒜=ℬ⁢(a), we get

(2.11) $${∥W_{a}\,\left(Q_{a}\,y,s\right)-w_{ℬ\,\left(a\right)}\,\left(\bar{y},s\right)∥}_{L_{\rho }^{2}}=o\,\left(\frac{1}{s^{2}}\right)\;\text{as }\,s\to +\infty .$$

Note that an alternative application of Proposition 2.1 with $W_{1}=W_{a}$ and $W_{2}=w_{ℬ\,\left(a\right)}$ yields either (2.5) or (2.6). However, the case (2.5) is excluded by (2.11). Hence, (2.8) follows. Since we showed in Corollary 2.2 that a↦ℬ⁢(a) is continuous, the same holds for a↦𝒜⁢(a).

As for (2.9), it is a direct consequence of the following lemma which allows us to carry estimate (2.8) from compact sets |y|≤K to sets $\left|y\right|\leq K\,\sqrt{s}$.

Lemma 2.6 (Extension of the Convergence from Compact Sets to Sets $\left|y\right|\leq K\,\sqrt{s}$)

*Assume that *Z* satisfies*

(2.12) $$\partial_{s}Z\leq \Delta \,Z-\frac{1}{2}\,y\cdot \nabla Z+Z+\frac{C_{1}}{s}\,Z,0\leq Z\,\left(y,s\right)\leq C_{1}\;\text{for all }\,\left(y,s\right)\in {\mathbb{R}}^{N}\times \left[\hat{s},+\infty \right),$$

*for some $C_{1}>0$. Then for all $s^{\prime }\geq \hat{s}$ and $s\geq s^{\prime }+1$ such that $e^{\left(s-s^{\prime }\right)/2}=\sqrt{s}$, we have*

$$\underset{\left|y\right|\leq K\,\sqrt{s}}{\sup}Z\,\left(y,s\right)\leq C\,\left(C_{1},K\right)\,e^{s-s^{\prime }}\,{∥Z\,\left(s^{\prime }\right)∥}_{L_{\rho }^{2}}.$$

**Proof.**

This lemma is a corollary of [15, Proposition 2.1] and it is proved in the course of the proof of [4, Proposition 2.13] (in particular, pp. 1203–1205). ∎

Let us derive (2.9) from Lemma 2.6. If we define $G\,\left(y,s\right)=W_{a}\,\left(Q_{a}\,y,s\right)-w_{ℬ\,\left(a\right)}\,\left(\bar{y},s\right)$, straightforward calculations based on (1.13) yield

(2.13) $$\partial_{s}G=\Delta \,G-\frac{1}{2}\,y\cdot \nabla G+G+\alpha \,G\;\text{for all }\,\left(y,s\right)\in {\mathbb{R}}^{N}\times \left[-\log T,+\infty \right),$$

where

$$\alpha \,\left(y,s\right)=\frac{{\left|W_{a}\right|}^{p-1}\,W_{a}-{\left|w_{ℬ}\right|}^{p-1}\,w_{ℬ}}{W_{a}-w_{ℬ}}-\frac{p}{p-1}=p\,{\left|\tilde{w}\,\left(y,s\right)\right|}^{p-1}-\frac{p}{p-1}\;\text{if }\,W_{a}\neq w_{ℬ},$$

for some $\tilde{w}\,\left(y,s\right)\in \left(W_{a}\,\left(Q_{a}\,y,s\right),w_{ℬ\,\left(a\right)}\,\left(\bar{y},s\right)\right)$.

From [11, Theorem 1], we know that for *s* large enough,

$${∥\tilde{w}\,\left(s\right)∥}_{L^{\infty }}\leq \kappa +\frac{C}{s},$$

which implies

(2.14) $$\alpha \,\left(y,s\right)\leq p\,{\left(\kappa +\frac{C}{s}\right)}^{p-1}-\frac{p}{p-1}\leq \frac{C_{1}}{s}.$$

If Z=|G|, then we use Kato’s inequality Δ⁢G⋅sgn⁡(G)≤Δ⁢(|G|) to derive equation (2.12) from (2.13) and (2.14). Applying Lemma 2.6 together with estimate (2.8) yields

$$\underset{\left|y\right|\leq K\,\sqrt{s}}{\sup}Z\,\left(y,s\right)\leq C\,e^{s-s^{\prime }}\,e^{-\frac{s^{\prime }}{2}}\,{\left(s^{\prime }\right)}^{C_{0}}\leq C\,e^{-\frac{s}{2}}\,s^{\frac{3}{2}+C_{0}}$$

for all $s^{\prime }\geq s_{1}$ and $s\geq s^{\prime }+1$ for some $s_{1}>0$ large such that $e^{\left(s-s^{\prime }\right)/2}=\sqrt{s}$. This yields (2.9). Estimate (2.10) directly follows from (2.9) by the transformation (1.12). This ends the proof of Proposition 2.5. ∎

### 2.2 Part 2: A Geometric Constraint Linked to the Asymptotic Behaviors

In this subsection, we follow the idea of [20] to introduce local ${𝒞}^{1,\alpha^{*}}$-charts of the blow-up set, and get a geometric constraint mechanism on the blow-up set (see Proposition 2.7 below) which is a crucial step in linking refined asymptotic behaviors of the solution to geometric descriptions of the blow-up set.

Consider $a\in S_{\delta }$ and ℓ∈{1,…,N-1}. We introduce the local ${𝒞}^{1,\alpha^{*}}$-chart of the blow-up set at the point *a* as follows:

$${\mathbb{R}}^{N-\ell }\to {\mathbb{R}}^{N},\tilde{\xi }\mapsto \left(\gamma_{a,1}\,\left(\tilde{\xi }\right),\ldots ,\gamma_{a,\ell }\,\left(\tilde{\xi }\right),\tilde{\xi }\right),$$

where $\tilde{\xi }=\left(\xi_{\ell +1},\ldots ,\xi_{N}\right)$ and $\gamma_{a,i}\in {𝒞}^{1,\alpha^{*}}\,\left({\left(-\epsilon_{a},\epsilon_{a}\right)}^{N-\ell }\right)$ for some $\alpha^{*}\in \left(0,\frac{1}{2}\right)$ and $\epsilon_{a}>0$. Then the set $S_{\delta }$ is locally near *a* defined by

(2.15) $$\left\{a+\sum_{i=1}^{\ell }\gamma_{a,i}\,\left(\tilde{\xi }\right)\,\eta_{i}\,\left(a\right)+\sum_{j=\ell +1}^{N}\xi_{k}\,\tau_{k}\,\left(a\right)|\left|\tilde{\xi }\right|<\epsilon_{a}\right\},$$

where $\eta_{1}\,\left(a\right),\ldots ,\eta_{\ell }\,\left(a\right)$ and $\tau_{\ell +1}\,\left(a\right),\ldots ,\tau_{N}\,\left(a\right)$ are of norm 1 and, respectively, normal and tangent to $S_{\delta }$ at *a*. By definition, we have

$$\gamma_{a,i}\,\left(0\right)=0\;\text{and}\;\nabla \gamma_{a,i}\,\left(0\right)=0\;\text{for all }\,i=1,\ldots ,\ell .$$

Let $Q_{a}$ be the orthogonal matrix whose columns are $\eta_{i}\,\left(a\right)$ and $\tau_{j}\,\left(a\right)$, namely

(2.16) $$\eta_{i}\,\left(a\right)=Q_{a}\,e_{i}\;\text{and}\;\tau_{j}\,\left(a\right)=Q_{a}\,e_{j}.$$

Define

(2.17) $$w_{a}\,\left(y,s\right)={\left(T-t\right)}^{\frac{1}{p-1}}\,u\,\left(x,t\right),y=Q_{a}^{T}\,\left(\frac{x-a}{\sqrt{T-t}}\right),s=-\log\left(T-t\right).$$

Then we see from (1.12) that $w_{a}$ satisfies (1.13) and

(2.18) $$w_{a}\,\left(y,s\right)=W_{a}\,\left(Q_{a}\,y,s\right)\;\text{for all }\,\left(y,s\right)\in {\mathbb{R}}^{N}\times \left[-\log T,+\infty \right).$$

Note from (2.16) that the point (y,s) in the domain of $w_{a}$ becomes the point (x,t) in the domain of *u*, where

$$x=a+e^{-\frac{s}{2}}\,Q_{a}\,y=a+e^{-\frac{s}{2}}\,\left(\sum_{i=1}^{\ell }y_{i}\,\eta_{i}\,\left(a\right)+\sum_{j=\ell +1}^{N}y_{j}\,\tau_{j}\,\left(a\right)\right),t=T-e^{-s}.$$

Now, fix $a\in S_{\delta }$ and consider an arbitrary $b\in S_{\delta }$. From (2.17), we have

(2.19) $$w_{a}\,\left(y,s\right)=w_{b}\,\left(Y,s\right),\text{where }\,Y=Q_{b}^{T}\,\left(Q_{a}\,y+e^{\frac{s}{2}}\,\left(a-b\right)\right).$$

If we differentiate (2.19) with respect to $y_{k}$ with k∈{ℓ+1,…,N}, we get

(2.20) $${\left(T-t\right)}^{\frac{1}{p-1}+\frac{1}{2}}\,\frac{\partial u}{\partial \tau_{k}\,\left(a\right)}\,\left(x,t\right)=\frac{\partial w_{a}}{\partial y_{k}}\,\left(y,s\right)=\sum_{i=1}^{\ell }\tau_{k}\,\left(a\right)\cdot \eta_{i}\,\left(b\right)\,\frac{\partial w_{b}}{\partial y_{i}}\,\left(Y,s\right)+\sum_{j=\ell +1}^{N}\tau_{k}\,\left(a\right)\cdot \tau_{j}\,\left(b\right)\,\frac{\partial w_{b}}{\partial y_{j}}\,\left(Y,s\right).$$

If we fix *b* as the projection of $x=a+e^{-\frac{s}{2}}\,Q_{a}\,y$ on the blow-up set in the orthogonal direction to the tangent space to the blow-up set at *a*, then *b* has the same components on the tangent space spanned by $\left\{\tau_{\ell +1}\,\left(a\right),\ldots ,\tau_{N}\,\left(a\right)\right\}$ as *x*. In particular,

(2.21) $$b=b\,\left(a,y,s\right)=a+\sum_{i=1}^{\ell }\gamma_{a,i}\,\left(e^{-\frac{s}{2}}\,\tilde{y}\right)\,\eta_{i}\,\left(a\right)+\sum_{j=\ell +1}^{N}e^{-\frac{s}{2}}\,y_{j}\,\tau_{j}\,\left(a\right),\tilde{y}=\left(y_{\ell +1},\ldots ,y_{N}\right).$$

The following proposition gives a geometric constraint on the expansion of $w_{a}$, which is the bridge linking the refined asymptotic behavior to the refined regularity of the blow-up set.

Proposition 2.7 (A Geometric Constraint on the Expansion of $w_{a}$)

*Assume that*

$$\gamma_{a}\in {𝒞}^{1,\alpha^{*}}\,\left({\left(-\epsilon_{a},\epsilon_{a}\right)}^{N-\ell },{\mathbb{R}}^{\ell }\right)\;\text{for some }\,\alpha^{*}\in \left(0,\frac{1}{2}\right)\,\text{ and }\,\epsilon_{a}>0.$$

*Then, there exists $s_{1}\geq \max\left\{-\log T,s_{0}\right\}$ ($s_{0}$ is introduced in Proposition 2.5) such that for all $a\in S_{\delta }$, |y|≤1, $s\geq s_{1}$ and k=ℓ+1,…,N, it holds that*

$$\left|\frac{\partial w_{a}}{\partial y_{k}}\,\left(y,s\right)-\left\{\frac{\partial w_{b}}{\partial y_{k}}\,\left(\bar{y},0,\ldots ,0,s\right)+\frac{\kappa }{2\,p\,s}\,\sum_{i=1}^{\ell }\frac{\partial \gamma_{a,i}}{\partial \xi_{k}}\,\left(e^{-\frac{s}{2}}\,\tilde{y}\right)\,y_{i}\right\}\right|$$

(2.22) $$\leq C\,\sum_{i=1}^{\ell }\left|\frac{\partial \gamma_{a,i}}{\partial \xi_{k}}\,\left(e^{-\frac{s}{2}}\,\tilde{y}\right)\right|\,\left[\left|\bar{y}\right|\,\frac{\log s}{s^{2}}+\frac{1}{s}\,e^{-\frac{\alpha^{*}\,s}{2}}+e^{-\frac{s}{2}}\,s^{C_{0}}\right]+C\,e^{-\frac{\left(1+\alpha^{*}\right)\,s}{2}}\,s^{C_{0}},$$

*where $\bar{y}=\left(y_{1},\ldots ,y_{\ell }\right)$, $\tilde{y}=\left(y_{\ell +1},\ldots ,y_{N}\right)$ and *b* is defined by (2.21).*

**Proof.**

Note that the proof of Proposition 2.7 was given in [20] only when ℓ=1. Of course, that proof naturally extends to the case when ℓ∈{2,…,N-1}. Since our paper is relevant only when ℓ≥2 and Proposition 2.7 presents an essential link between the asymptotic behavior of the solution and a geometric constraint of the blow-up set, we felt we should give the proof of this proposition for the completeness and for the reader’s convenience. As said earlier, this section just gives the main steps of the proof of Theorem 1.1, and because the proof is long and technical, we leave it to Section 3.3. ∎

### 2.3 Part 3: Refined Regularity of the Blow-Up Set and Conclusion of Theorem 1.1

In this subsection, we give the proof of the ${𝒞}^{2}$-regularity of the blow-up set (Theorems 1.1 and 1.3). We proceed in two steps:

• Step 1: We derive from Proposition 2.5 that $\gamma_{a}$ is ${𝒞}^{1,\frac{1}{2}-\eta }$ for all η>0. Then we apply Proposition 2.7 with $\alpha^{*}=\alpha \in \left(0,\frac{1}{2}\right)$ to improve the regularity of $\gamma_{a}$ which reaches ${𝒞}^{1,1-\eta }$ for all η>0.
• Step 2: Using the ${𝒞}^{1,1-\eta }$-regularity and the geometric constraint in Proposition 2.7, we refine the asymptotic behavior given in Proposition 2.5, which involves terms of order $\frac{1}{s}\,e^{-\frac{s}{2}}$. Exploiting this refined asymptotic behavior together with the geometric constraint (2.22), we derive that $\gamma_{a}$ is of class ${𝒞}^{2}$, which is the conclusion of Theorem 1.1. From the information obtained on the ${𝒞}^{2}$-regularity, we calculate the second fundamental form of the blow-up set, which concludes the proof of Theorem 1.3.

#### Step 1: Deriving ${𝒞}^{1,1-\eta }$-Regularity of the Blow-Up Set.

We first derive the ${𝒞}^{1,\frac{1}{2}-\eta }$-regularity of the blow-up set for all η>0 from Proposition 2.5. Then we apply Proposition 2.7 with $\alpha^{*}=\alpha \in \left(0,\frac{1}{2}\right)$ to get ${𝒞}^{1,1-\eta }$-regularity for all η>0. In particular, we claim the following:

Proposition 2.8 ($C^{1,\frac{1}{2}-\eta }$-Regularity for *S*)

*Under the hypotheses of Theorem 1.1, *S* is the graph of a vector function $\gamma \in C^{1,\frac{1}{2}-\eta }\,\left({\left(-\delta_{1},\delta_{1}\right)}^{N-\ell },R^{\ell }\right)$ for any η>0, locally near $\hat{a}$. More precisely, there is an $h_{0}>0$ such that for all $\left|\tilde{\xi }\right|<\delta_{1}$ and $\left|\tilde{h}\right|<h_{0}$ such that $\left|\tilde{\xi }+\tilde{h}\right|<\delta_{1}$, one has for all i∈{1,…,ℓ},*

(2.23) $$\left|\gamma_{i}\,\left(\tilde{\xi }+\tilde{h}\right)-\gamma_{i}\,\left(\tilde{\xi }\right)-\tilde{h}\cdot \nabla \gamma_{i}\,\left(\tilde{\xi }\right)\right|\leq C\,{\left|\tilde{h}\right|}^{\frac{3}{2}}\,{\left|\log\left|\tilde{h}\right|\right|}^{\frac{1}{2}+\frac{C_{0}}{2}}.$$

**Proof.**

The proof is mainly based on the derivation of the sharp asymptotic profile given in Proposition 2.5. In fact, we exploit the estimate (2.10) to find out a geometric constraint on the blow-up set *S*, which implies some more regularity on *S*. Since the argument follows the same lines as in [17, Section 4] for the case ℓ=1, and no new ideas are needed for the case ℓ≥2, we will just sketch the proof by underlying the most relevant aspects in Section 3.2 for the sake of convenience. ∎

The next proposition shows the ${𝒞}^{1,1-\eta }$-regularity of the blow-up set.

Proposition 2.9 ($C^{1,1-\eta }$-Regularity for $S_{\delta }$)

*There exists $\xi_{0}>0$ such that for each $a\in S_{\delta }$, the local chart defined in (2.15) satisfies for all k=ℓ+1,…,N and $\left|\tilde{\xi }\right|<\xi_{0}$,*

$$\sum_{i=1}^{\ell }\left|\frac{\partial \gamma_{a,i}}{\partial \xi_{k}}\,\left(\tilde{\xi }\right)\right|\leq C\,\left|\tilde{\xi }\right|\,{\left|\log\left|\tilde{\xi }\right|\right|}^{1+\mu }\;\text{for some }\,\mu >0.$$

**Proof.**

Note that the case ℓ=1 was already proven in [20, p. 516, Lemma 3.4]. Here we use again the argument of [20] for the case ℓ≥2. Using the estimate given in Proposition 2.5 and parabolic regularity, we see that for all k≥ℓ+1 and $s\geq s_{0}+1$,

$$\underset{a\in S_{\delta },\left|y\right|<2}{\sup}\left|\frac{\partial w_{a}}{\partial y_{k}}\,\left(y,s\right)\right|\leq C\,e^{-\frac{s}{2}}\,s^{\mu }\;\text{for some }\,\mu >0.$$

Consider $a\in S_{\delta }$ and $y=\left(\bar{y},\tilde{y}\right)$, where $\bar{y}=\left(y_{1},\ldots ,y_{\ell }\right)$ is such that $y_{i_{*}}=1$ for some $i_{*}\in \left\{1,\ldots ,\ell \right\}$, $y_{j}=0$ for $1\leq j\neq i_{*}\leq \ell$, and $\tilde{y}=\left(y_{\ell +1},\ldots ,y_{N}\right)$ is arbitrary in $\partial B_{N-\ell }\,\left(0,1\right)$. For $s\geq \max\left\{s_{0}+1,s_{1}\right\}$, we consider b=b⁢(a,y,s) defined as in (2.21). Since $\gamma_{a}$ is ${𝒞}^{1,\frac{1}{2}-\eta }$ for any η>0, we use (2.22) with $\alpha^{*}=\alpha \in \left(0,\frac{1}{2}\right)$ to write for k∈{ℓ+1,…,N},

$$\frac{\kappa }{2\,p\,s}\,\left|\frac{\partial \gamma_{a,i_{*}}}{\partial \xi_{k}}\,\left(e^{-\frac{s}{2}}\,\tilde{y}\right)\right|\leq C\,\frac{\log s}{s^{2}}\,\sum_{i=1}^{\ell }\left|\frac{\partial \gamma_{a,i}}{\partial \xi_{k}}\,\left(e^{-\frac{s}{2}}\,\tilde{y}\right)\right|+C\,e^{-\frac{s}{2}}\,s^{\mu }.$$

Since $i_{*}$ is arbitrary in {1,…,ℓ}, we get

$$\frac{\kappa }{2\,p\,s}\,\sum_{i=1}^{\ell }\left|\frac{\partial \gamma_{a,i}}{\partial \xi_{k}}\,\left(e^{-\frac{s}{2}}\,\tilde{y}\right)\right|\leq C\,\frac{\log s}{s^{2}}\,\sum_{i=1}^{\ell }\left|\frac{\partial \gamma_{a,i}}{\partial \xi_{k}}\,\left(e^{-\frac{s}{2}}\,\tilde{y}\right)\right|+C\,e^{-\frac{s}{2}}\,s^{\mu },$$

which gives

$$\sum_{i=1}^{\ell }\left|\frac{\partial \gamma_{a,i}}{\partial \xi_{k}}\,\left(e^{-\frac{s}{2}}\,\tilde{y}\right)\right|\leq C\,e^{-\frac{s}{2}}\,s^{1+\mu }.$$

If $\tilde{\xi }=e^{-\frac{s}{2}}\,\tilde{y}$, then $\left|\tilde{\xi }\right|=e^{-\frac{s}{2}}$ and $\left|\log\left|\tilde{\xi }\right|\right|=\frac{s}{2}$ since $\left|\tilde{y}\right|=1$. Therefore,

$$\sum_{i=1}^{\ell }\left|\frac{\partial \gamma_{a,i}}{\partial \xi_{k}}\,\left(e^{-\frac{s}{2}}\,\tilde{y}\right)\right|\leq C\,\left|\tilde{\xi }\right|\,{\left|\log\left|\tilde{\xi }\right|\right|}^{1+\mu }.$$

Since $\tilde{y}$ is arbitrary in $\partial B_{N-\ell }\,\left(0,1\right)$, $\tilde{\xi }=e^{-\frac{s}{2}}\,\tilde{y}$ covers a whole neighborhood of 0, namely $B\,\left(0,\xi_{0}\right)$, where $\xi_{0}=e^{-\frac{1}{2}\,\max\left\{s_{0}+1,s_{1}\right\}}$. This concludes the proof of Proposition 2.9. ∎

#### Step 2: Further Refined Asymptotic Behavior and Deriving ${𝒞}^{2}$-Regularity of *S*.

In this part, we shall use the ${𝒞}^{1,1-\eta }$-regularity of the blow-up set together with the geometric constraint (2.22) in order to refine further the asymptotic behavior (2.8). In particular, we claim the following:

Proposition 2.10 **(Further Refined Asymptotic Behavior (2.8))**

*There exist $s_{2}>0$, $d\in \left(0,\frac{1}{2}\right)$ and continuous functions $a\to \lambda_{\beta }\,\left(a\right)$ for all $\beta \in N^{N}$ with |β|=3 and $\left|\bar{\beta }\right|=1$, where $\bar{\beta }=\left(\beta_{1},\ldots ,\beta_{\ell }\right)$, $\left|\bar{\beta }\right|=\sum_{i=1}^{\ell }\beta_{i}$, such that for all $a\in S_{\delta }$ and $s\geq s_{2}$,*

(2.24) $${∥W_{a}\,\left(Q_{a}\,y,s\right)-w_{ℬ\,\left(a\right)}\,\left(\bar{y},s\right)-\frac{e^{-\frac{s}{2}}}{s}\,\sum_{\left|\beta \right|=3,\left|\bar{\beta }\right|=1}\lambda_{\beta }\,\left(a\right)\,h_{\beta }\,\left(y\right)∥}_{L_{\rho }^{2}}\leq C\,e^{-\frac{s}{2}}\,s^{d-\frac{3}{2}},$$

*where $h_{\beta }$ is defined in (3.2).*

**Proof.**

The proof of this proposition is based on ideas of [20] where the case ℓ=1 was treated. As in [20], the geometric constraint given in Proposition 2.7 plays an important role in deriving (2.24). Since the proof is long and technical, we leave it to Section 3.4. ∎

Let us derive Theorem 1.1 from Propositions 2.10 and 2.7. In particular, Theorem 1.1 is a direct consequence of the following result.

Proposition 2.11 *For all $a\in S_{\delta }$, we have for all i∈{1,…,ℓ}, j,k∈{ℓ+1,…,N},*

$$\Lambda_{j,k}^{\left(i\right)}\,\left(a\right)=\frac{\partial^{2}\gamma_{a,i}}{\partial \xi_{j}\,\xi_{k}}\,\left(0\right)=\frac{2\,p}{\kappa }\,\left(1+\delta_{j,k}\right)\,\lambda_{e_{i}+e_{j}+e_{k}}\,\left(a\right),$$

*where $a\to \lambda_{\beta }\,\left(a\right)$ is introduced in Proposition 2.10, $e_{i}$ is the *i*-th vector of canonical base of $R^{N}$, and $\delta_{i,k}$ is the Kronecker symbol. *

**Proof.**

From (2.18), (2.24) and the fact that estimate (2.24) also holds in $W^{2,\infty }\left(|y|<2\right)$ by parabolic regularity, we derive for all k≥ℓ+1 and $s\geq s_{2}+1$,

(2.25) $$\underset{a\in S_{\delta },\left|y\right|<2}{\sup}\left|\frac{\partial w_{a}}{\partial y_{k}}\,\left(y,s\right)-\frac{e^{-\frac{s}{2}}}{s}\,\sum_{\left|\beta \right|=3,\left|\bar{\beta }\right|=1}\lambda_{\beta }\,\left(a\right)\,\frac{\partial h_{\beta }}{\partial y_{k}}\,\left(y\right)\right|\leq C\,e^{-\frac{s}{2}}\,s^{d-\frac{3}{2}},$$

for some $d\in \left(0,\frac{1}{2}\right)$. Note that if $\left|\bar{\beta }\right|=1$, then there is a unique index $i^{*}\in \left\{1,\ldots ,\ell \right\}$ such that $\beta_{i^{*}}=1$ and $\beta_{m}=0$ for m∈{1,…,ℓ}, $m\neq i^{*}$. Note also from the definition of $h_{\beta }$ (see (3.2) below) that

$$\frac{\partial h_{\beta }}{\partial y_{k}}\,\left(y\right)=\beta_{k}\,h_{\beta_{k}-1}\,\left(y_{k}\right)\,\prod_{j=1,j\neq k}^{N}h_{\beta_{j}}\,\left(y_{j}\right),$$

and that $h_{0}=1$. Therefore, (2.25) yields

(2.26) $$\left|\frac{\partial w_{a}}{\partial y_{k}}\,\left(y,s\right)-\frac{e^{-\frac{s}{2}}}{s}\,\sum_{i=1}^{\ell }\sum_{\left|\beta \right|=3,\beta_{i}=1}\lambda_{\beta }\,\left(a\right)\,h_{1}\,\left(y_{i}\right)\,\beta_{k}\,h_{\beta_{k}-1}\,\left(y_{k}\right)\,\prod_{j=\ell_{a}+1,j\neq k}h_{\beta_{j}}\,\left(y_{j}\right)\right|\leq C\,e^{-\frac{s}{2}}\,s^{d-\frac{3}{2}}.$$

Take $i^{*}\in \left\{1,\ldots ,\ell \right\}$ arbitrarily and $y=e_{i^{*}}+\epsilon \,e_{j}$ where ϵ=±1 and j≥ℓ+1. Since $h_{m}\,\left(0\right)=0$ if *m* is odd, and $\beta_{i^{*}}=1$ if |β|=3, we have either $\beta =e_{i^{*}}+e_{j^{*}}+e_{k^{*}}$ or $\beta =e_{i^{*}}+2\,e_{j^{*}}$ for some $j^{*},k^{*}\in \left\{\ell +1,\ldots ,N\right\}$. Using (2.26) yields

(2.27) $$\left|\frac{\partial w_{a}}{\partial y_{k}}\,\left(e_{i^{*}}+\epsilon \,e_{j},s\right)-\epsilon \,\frac{e^{-\frac{s}{2}}}{s}\,\left(1+\delta_{k,j}\right)\,\lambda_{e_{i^{*}}+e_{k}+e_{j}}\,\left(a\right)\right|\leq C\,e^{-\frac{s}{2}}\,s^{d-\frac{3}{2}}.$$

Similarly, we have

(2.28) $$\left|\frac{\partial w_{a}}{\partial y_{k}}\,\left(e_{i^{*}},s\right)\right|\leq C\,e^{-\frac{s}{2}}\,s^{d-\frac{3}{2}}.$$

Now using Proposition 2.7, we write for $y=e_{i^{*}}+\epsilon \,e_{j}$ and $s\geq \max\left\{s_{2}+1,s_{1}\right\}$,

$$\left|\frac{\partial w_{a}}{\partial y_{k}}\,\left(e_{i^{*}}+\epsilon \,e_{j},s\right)-\frac{\partial w_{a}}{\partial y_{k}}\,\left(e_{i^{*}},s\right)-\frac{\kappa }{2\,p\,s}\,\frac{\partial \gamma_{a,i^{*}}}{\partial \xi_{k}}\,\left(e^{-\frac{s}{2}}\,\epsilon \,e_{j}\right)\right|$$

$$\leq C\,\frac{\log s}{s^{2}}\,\sum_{i=1}^{\ell }\left|\frac{\partial \gamma_{a,i}}{\partial \xi_{k}}\,\left(e^{-\frac{s}{2}}\,\epsilon \,e_{j}\right)\right|+C\,e^{-\frac{\left(1+\alpha^{*}\right)\,s}{2}}\,s^{C_{0}}+C\,e^{-s}\,s^{C_{0}+1}.$$

Using this estimate together with (2.27) and (2.28), we obtain

(2.29) $$\left|\epsilon \,e^{-\frac{s}{2}}\,\left(1+\delta_{k,j}\right)\,\lambda_{e_{i^{*}}+e_{k}+e_{j}}\,\left(a\right)-\frac{\kappa }{2\,p}\,\frac{\partial \gamma_{a,i^{*}}}{\partial \xi_{k}}\,\left(e^{-\frac{s}{2}}\,\epsilon \,e_{j}\right)\right|\leq C\,\frac{\log s}{s}\,\sum_{i=1}^{\ell }\left|\frac{\partial \gamma_{a,i}}{\partial \xi_{k}}\,\left(e^{-\frac{s}{2}}\,\epsilon \,e_{j}\right)\right|+C\,e^{-\frac{s}{2}}\,s^{d-\frac{1}{2}}.$$

From Proposition 2.10, we see that

$${∥W_{a}\,\left(Q_{a}\,y,s\right)-w_{ℬ\,\left(a\right)}\,\left(\bar{y},s\right)∥}_{L_{\rho }^{2}}\leq C\,s^{-1}\,e^{-\frac{s}{2}}\;\text{for all }\,s\geq s_{2}.$$

Using this estimate and noticing that the same proof of Proposition 2.9 holds with μ=-1, we derive

$$\sum_{i=1}^{\ell }\left|\frac{\partial \gamma_{a,i}}{\partial \xi_{k}}\,\left(e^{-\frac{s}{2}}\,\epsilon \,e_{j}\right)\right|\leq C\,e^{-\frac{s}{2}}.$$

Putting this estimate into (2.29) and noticing that $\frac{\partial \gamma_{a,i^{*}}}{\partial \xi_{k}}\,\left(0\right)=0$, we find that

(2.30) $$\frac{\partial^{2}\gamma_{a,i^{*}}}{\partial \xi_{k}\,\partial \xi_{j}}\,\left(0\right)=\lim_{s\to +\infty }\frac{\frac{\partial \gamma_{a,i^{*}}}{\partial \xi_{k}}\,\left(e^{-\frac{s}{2}}\,\epsilon \,e_{j}\right)}{\epsilon \,e^{-\frac{s}{2}}}=\frac{2\,p}{\kappa }\,\left(1+\delta_{k,j}\right)\,\lambda_{e_{i^{*}}+e_{k}+e_{j}}\,\left(a\right).$$

Since $i^{*}$ is taken arbitrarily belonging to {1,…,ℓ}, identity (2.30) holds for all $i^{*}\in \left\{1,\ldots ,\ell \right\}$. This concludes the proof of Proposition 2.11. ∎

**Proof of Theorem 1.1.**

From the definition of the local chart (2.15), we have $\gamma_{a,i}\,\left(0\right)=\nabla \gamma_{a,i}\,\left(0\right)=0$ for all i∈{1,…,ℓ}. Hence, we deduce from (2.30) the expression of the second fundamental form of the blow-up set at the point *a* along the unitary basic vector $Q_{a}^{T}\,e_{i}$: for all k,j∈{ℓ+1,…,N},

(2.31) $$\Lambda_{k,j}^{\left(i\right)}\,\left(a\right)=\frac{\partial^{2}\gamma_{a,i}}{\partial \xi_{k}\,\partial \xi_{j}}\,\left(0\right)=\frac{2\,p}{\kappa }\,\left(1+\delta_{k,j}\right)\,\lambda_{e_{i}+e_{k}+e_{j}}\,\left(a\right).$$

In addition, since $a\to \lambda_{\beta }\,\left(a\right)$ is continuous, we conclude that the blow-up set is of class ${𝒞}^{2}$. This completes the proof of Theorem 1.1. ∎

**Proof of Theorem 1.3.**

The estimate (1.23) directly follows from Propositions 2.10 and 2.11. Indeed, the sum in estimate (2.24) can be indexed as

$$\left\{\beta \in {\mathbb{N}}^{N}|\left|\beta \right|=3,\left|\bar{\beta }\right|=1\right\}=\left\{e_{i}+e_{j}+e_{k}|1\leq i\leq \ell ,\ell +1\leq j,k\leq N\right\},$$

where $e_{k}$ is the *k*-th canonical basis vector of ${\mathbb{R}}^{N}$. By (2.31) and the definition of $h_{\beta }$ (see (3.2) below), we write

$$\sum_{\left|\beta \right|=3,\left|\bar{\beta }\right|=1}\lambda_{\beta }\,\left(a\right)\,h_{\beta }\,\left(y\right)=\sum_{i=1}^{\ell }\sum_{j,k=\ell +1}^{N}\lambda_{e_{i}+e_{j}+e_{k}}\,h_{e_{i}+e_{j}+e_{k}}\,\left(y\right)$$

$$=\frac{\kappa }{2\,p}\,\sum_{i=1}^{\ell }y_{i}\,\sum_{j,k=\ell +1}^{N}\frac{\Lambda_{j,k}^{\left(i\right)}\,\left(a\right)}{1+\delta_{j,k}}\,\left(y_{j}\,y_{k}-2\,\delta_{j,k}\right),$$

which yields (1.23).

As for (1.24), we note from (2.24) that for all |β|=3 with $\left|\bar{\beta }\right|=1$, one has

$$\left|g_{a,\beta }\,\left(s\right)-\frac{e^{-\frac{s}{2}}}{s}\,\lambda_{\beta }\,\left(s\right)\right|\leq C\,e^{-\frac{s}{2}}\,s^{d-\frac{3}{2}}$$

(recall that $g_{a}\,\left(y,s\right)=W_{a}\,\left(Q_{a}\,y,s\right)-w_{ℬ\,\left(a\right)}\,\left(\bar{y},s\right)$). Hence, we write from (2.31),

$$\Lambda_{j,k}^{\left(i\right)}\,\left(a\right)=\frac{2\,p}{\kappa }\,\left(1+\delta_{j,k}\right)\,\lambda_{e_{i}+e_{j}+e_{k}}\,\left(a\right)$$

$$=\frac{2\,p}{\kappa }\,\left(1+\delta_{j,k}\right)\,\lim_{s\to +\infty }s\,e^{\frac{s}{2}}\,g_{a,e_{i}+e_{j}+e_{k}}\,\left(s\right)$$

$$=\frac{2\,p}{\kappa }\,\left(1+\delta_{j,k}\right)\,\lim_{s\to +\infty }s\,e^{\frac{s}{2}}\,\int_{{\mathbb{R}}^{N}}g_{a}\,\left(y,s\right)\,\frac{h_{e_{i}+e_{j}+e_{k}}\,\left(y\right)}{{∥h_{e_{i}+e_{j}+e_{k}}∥}_{L_{\rho }^{2}}^{2}}\,\rho \,\left(y\right)\,𝑑y.$$

Using again the definition of $h_{\beta }$ (see (3.2) below), we see that

$$h_{e_{i}+e_{j}+e_{k}}=y_{i}\,\left(y_{j}\,y_{k}-\delta_{j,k}\right)\;\text{and}\;{∥h_{e_{i}+e_{j}+e_{k}}∥}_{L_{\rho }^{2}}^{2}=8\,\left(1+\delta_{j,k}\right).$$

Recall that $w_{𝒜}$ does not depend on $y_{j}$ for j≥ℓ+1. Hence, for all j,k≥ℓ+1,

$$\Lambda_{j,k}^{\left(i\right)}\,\left(a\right)=\frac{p}{4\,\kappa }\,\lim_{s\to +\infty }s\,e^{\frac{s}{2}}\,\int_{{\mathbb{R}}^{N}}W_{a}\,\left(Q_{a}\,y,s\right)\,y_{i}\,\left(y_{j}\,y_{k}-2\,\delta_{j,k}\right)\,\rho \,\left(y\right)\,𝑑y,$$

which is (1.24). This concludes the proof of Theorem 1.3. ∎

## 3 Proof of Propositions 2.1, 2.7, 2.8 and 2.10

### 3.1 Classification of the Difference of Two Solutions of (1.13) Having the Same Asymptotic Behavior

In this subsection, we give the proof of Proposition 2.1. The formulation is the same as given in [4] for the difference of two solutions with the radial profile (ℓ=N). Therefore, we sketch the proof and emphasize only the novelties. Note also that the case ℓ=1 was treated in [17].

Let us define

$$g\,\left(y,s\right)=W_{1}\,\left(y,s\right)-W_{2}\,\left(y,s\right),$$

where $W_{i}$, i=1,2 are the solutions of equation (1.13) and behave like (2.3). We see from (1.13) and (2.3) that for all $\left(y,s\right)\in {\mathbb{R}}^{N}\times \left[-\log T,+\infty \right)$,

(3.1) $$\partial_{s}g=ℒ\,g+\alpha \,g,{∥g\,\left(s\right)∥}_{L_{\rho }^{2}}\leq C\,\frac{\log s}{s^{2}},$$

where

$$ℒ=\Delta -\frac{1}{2}\,y\cdot \nabla +1$$

and

$$\alpha \,\left(y,s\right)=\frac{{\left|W_{1}\right|}^{p-1}\,W_{1}-{\left|W_{2}\right|}^{p-1}\,W_{2}}{W_{1}-W_{2}}-\frac{p}{p-1}\;\text{if }\,W_{1}\neq W_{2},$$

in particular,

$$\alpha \,\left(y,s\right)=p\,{\left|W_{0}\,\left(y,s\right)\right|}^{p-1}-\frac{p}{p-1}\;\text{for some }\,W_{0}\,\left(y,s\right)\in \left(W_{1}\,\left(y,s\right),W_{2}\,\left(y,s\right)\right).$$

The operator ℒ is self-adjoint on $𝒟\,\left(ℒ\right)\subset L_{\rho }^{2}\,\left({\mathbb{R}}^{N}\right)$. Its spectrum consists of eigenvalues

$$\mathrm{spec}\left(ℒ\right)=\left\{\lambda_{n}=1-\frac{n}{2}|n\in \mathbb{N}\right\}.$$

The eigenfunctions corresponding to $1-\frac{n}{2}$ are

(3.2) $$h_{\beta }\,\left(y\right)=h_{\beta_{1}}\,\left(y_{1}\right)\,\cdots \,h_{\beta_{N}}\,\left(y_{N}\right),\beta_{1}+\cdots +\beta_{N}=\left|\beta \right|=n,$$

where

$$h_{m}\,\left(\xi \right)=\sum_{i=0}^{\left[m/2\right]}\frac{m!}{i!\,\left(m-2\,i\right)!}\,{\left(-1\right)}^{i}\,\xi^{m-2\,i},m\in \mathbb{N}$$

satisfy

$$\int_{\mathbb{R}}h_{m}\,\left(\xi \right)\,h_{n}\,\left(\xi \right)\,\rho \,\left(\xi \right)\,𝑑\xi =2^{m}\,m!\,\delta_{m,n}.$$

The component of *g* on $h_{\beta }$ is given by

$$g_{\beta }\,\left(s\right)=\int_{{\mathbb{R}}^{N}}k_{\beta }\,\left(y\right)\,g\,\left(y,s\right)\,\rho \,\left(y\right)\,𝑑y,\text{where }\,k_{\beta }\,\left(y\right)=\frac{h_{\beta }\,\left(y\right)}{{∥h_{\beta }∥}_{L_{\rho }^{2}}^{2}}.$$

If we denote by $P_{n}$ the orthogonal projector of $L_{\rho }^{2}$ over the eigenspace of ℒ corresponding to the eigenvalue $1-\frac{n}{2}$, then

$$P_{n}\,g\,\left(y,s\right)=\sum_{\left|\beta \right|=n}g_{\beta }\,\left(s\right)\,h_{\beta }\,\left(y\right).$$

Since the eigenfunctions of ℒ span the whole space $L_{\rho }^{2}$, we can write

$$g\,\left(y,s\right)=\sum_{n\in N}P_{n}\,g\,\left(y,s\right)=\sum_{\beta \in {\mathbb{N}}^{N}}g_{\beta }\,\left(s\right)\,h_{\beta }\,\left(y\right)=\sum_{\beta \in {\mathbb{N}}^{N},\left|\beta \right|\leq k}g_{\beta }\,\left(s\right)\,h_{\beta }\,\left(y\right)+R_{k+1}\,g\,\left(y,s\right),$$

where $R_{k}\,g=\sum_{n\geq k}P_{n}\,g$. We also denote

$$I\,{\left(s\right)}^{2}={∥g\,\left(s\right)∥}_{L_{\rho }^{2}}^{2}=\sum_{n\in \mathbb{N}}l_{n}^{2}\,\left(s\right)=\sum_{n\leq k}l_{n}^{2}\,\left(s\right)+r_{k+1}^{2}\,\left(s\right),$$

where

(3.3) $$l_{n}\,\left(s\right)={∥P_{n}\,g\,\left(s\right)∥}_{L_{\rho }^{2}},r_{k}\,\left(s\right)={∥R_{k}\,g\,\left(s\right)∥}_{L_{\rho }^{2}}.$$

As for α, we have the following estimates.

Lemma 3.1 **(Estimates on α)**

*For all $y\in R^{N}$ and s≥-log⁡T, we have*

$$\alpha \,\left(y,s\right)\leq \frac{C}{s},\left|\alpha \,\left(y,s\right)\right|\leq \frac{C}{s}\,\left(1+{\left|y\right|}^{2}\right),\left|\alpha \,\left(y,s\right)+\frac{1}{4\,s}\,\sum_{i=1}^{\ell }h_{2}\,\left(y_{i}\right)\right|\leq \frac{C}{s^{\frac{3}{2}}}\,\left(1+{\left|y\right|}^{3}\right).$$

**Proof.**

The proof follows the same lines as the proof of [4, Lemma 2.5] where the case ℓ=N was treated. ∎

In the following lemma, we project equation (3.1) on the different modes to get estimates for I⁢(s), $l_{n}\,\left(s\right)$ and $r_{n}\,\left(s\right)$. More precisely, we claim the following:

Lemma 3.2 (Evolution of I⁢(s), $l_{n}\,\left(s\right)$ and $r_{n}\,\left(s\right)$)

*There exist $s_{3}\geq -\log T$ and $s_{*}>0$ such that for all $s\geq s_{3}$, n∈N and $\beta \in N^{N}$, one has*

(3.4) $$\left|l_{n}^{\prime }\,\left(s\right)+\left(\frac{n}{2}-1\right)\,l_{n}\,\left(s\right)\right|\leq C\,\left(n\right)\,\frac{I\,\left(s\right)}{s},$$

(3.5) $$I^{\prime }\,\left(s\right)\leq \left(1-\frac{n+1}{2}+\frac{C_{0}}{s}\right)\,I\,\left(s\right)+\sum_{k=0}^{n}\frac{1}{2}\,\left(n+1-k\right)\,l_{k}\,\left(s\right),$$

(3.6) $$\left|g_{\beta }^{\prime }\,\left(s\right)+\left(-1+\frac{\left|\beta \right|}{2}+\frac{1}{s}\,\sum_{i=1}^{\ell }\beta_{i}\right)\,g_{\beta }\,\left(s\right)\right|\leq C\,\left(\beta \right)\,\left(\frac{1}{s^{\frac{3}{2}}}\,I\,\left(s\right)+\frac{1}{s}\,\left(l_{\left|\beta \right|-2}\,\left(s\right)+l_{\left|\beta \right|+2}\right)\right),$$

(3.7) $$r_{n}^{\prime }\,\left(s\right)\leq \left(1-\frac{n}{2}\right)\,r_{n}\,\left(s\right)+\frac{C}{s}\,I\,\left(s-s_{*}\right).$$

**Proof.**

See [4, Lemma 2.7] for (3.4) and (3.5). See [17, p. 545, Appendix B.1] for a calculation similar to (3.6). For (3.7), see [20, p. 523], where the calculation is mainly based on the following regularizing property of equation (3.1) by Herrero and Velázquez [9] (control of the $L_{\rho }^{4}$-norm by the $L_{\rho }^{2}$-norm up to some delay in time, see [9, Lemma 2.3]):

$${\left(\int g^{4}\,\left(y,s\right)\,\rho \,𝑑y\right)}^{\frac{1}{4}}\leq C\,{\left(\int g^{2}\,\left(y,s-s_{*}\right)\,\rho \,𝑑y\right)}^{\frac{1}{2}}\;\text{for some }\,s_{*}>0.$$

This ends the proof of Lemma 3.2. ∎

In the next step, we use Lemma 3.2 to show that either the null mode or a negative mode of ℒ will dominate as s→+∞. In particular, we have the following:

Proposition 3.3 **(Dominance of a Mode and Its Description)**

(i) *Either* $l_{n}\,\left(s\right)=𝒪\,\left(\frac{I\,\left(s\right)}{s}\right)$ *for all* n∈ℕ *, and there exist* $\sigma_{n}$ , $C_{n}>0$* and *$C_{n}^{\prime }>0$* such that*
$$I\,\left(s\right)\leq C_{n}\,s^{C_{n}^{\prime }}\,\exp\left(\left(1-\frac{n}{2}\right)\,s\right)\;\text{for all }\,s\geq \sigma_{n};$$
(ii) *or there is* $n_{0}\geq 2$ *such that*
(3.8) $$I\,\left(s\right)∼l_{n_{0}}\,\left(s\right)\;\text{𝑎𝑛𝑑}\;l_{n}\,\left(s\right)=𝒪\,\left(\frac{I\,\left(s\right)}{s}\right)\;\text{as }\,s\to +\infty \;\text{for all }\,n\neq n_{0}.$$

*Moreover, *

•
*if* $n_{0}=2$ *, namely* $I\,\left(s\right)∼l_{2}\,\left(s\right)$ *, then for all* |β|=2 ,
(3.9) $$\left\{ \begin{array}{cc} \left|g_{\beta }\,\left(s\right)\right|\leq C\,\frac{\log s}{s^{\frac{5}{2}}} & \text{if }\,\sum_{i=1}^{\ell }\beta_{i}\neq 2,\\ \left|g_{\beta }\,\left(s\right)-\frac{c_{\beta }}{s^{2}}\right|\leq C\,\frac{\log s}{s^{\frac{5}{2}}} & \text{if }\,\sum_{i=1}^{\ell }\beta_{i}=2, \end{array}\right.$$
•
*if* $n_{0}=3$ *, namely* $I\,\left(s\right)∼l_{3}\,\left(s\right)$ *, then*
(3.10) $$I\,\left(s\right)\leq C_{0}\,e^{-\frac{s}{2}}\,s^{C_{0}}\;\text{for some }\,C_{0}>0.$$

**Proof.**

See [4, Proposition 2.6] for the existence of a dominating component, where the proof relies on (3.4) and (3.5). If case (ii) occurs with $n_{0}=2$, by (3.6) we write for all $\beta \in {\mathbb{N}}^{N}$ with |β|=2,

$$\left|g_{\beta }^{\prime }\,\left(s\right)+\frac{g_{\beta }}{s}\,\sum_{i=1}^{\ell }\beta_{i}\right|\leq C\,\left(\beta \right)\,\left(\frac{I\,\left(s\right)}{s^{\frac{3}{2}}}+\frac{l_{0}\,\left(s\right)+l_{4}\,\left(s\right)}{s}\right)\leq C\,\left(\beta \right)\,\frac{I\,\left(s\right)}{s^{\frac{3}{2}}}\leq C\,\left(\beta \right)\,\frac{\log s}{s^{\frac{7}{2}}},$$

where we used (3.8) and (3.1) from which we have $l_{0}\,\left(s\right)+l_{4}\,\left(s\right)=𝒪\,\left(\frac{I\,\left(s\right)}{s}\right)$ and $I\,\left(s\right)=𝒪\,\left(\frac{\log s}{s^{2}}\right)$. Since $\sum_{i=1}^{\ell }\beta_{i}$ is only equal to 0,1 or 2 if |β|=2, estimate (3.9) follows after integration. Estimate (3.10) immediately follows from (3.4). This ends the proof of Proposition 3.3. ∎

Let us now derive Proposition 2.1 from Proposition 3.3. Indeed, we see from Proposition 3.3 that if case (i) occurs, we already have exponential decay for I⁢(s). If case (ii) occurs with $n_{0}\geq 3$, by (3.4) we write

$$\left|l_{n_{0}}^{\prime }\,\left(s\right)+\left(\frac{n_{0}}{2}-1\right)\,l_{n_{0}}\right|\leq \frac{C}{s}\,l_{n_{0}}.$$

Since $l_{n_{0}}\neq 0$ in a neighborhood of infinity, this gives

$$l_{n_{0}}\,\left(s\right)\leq C_{0}\,s^{C_{0}}\,e^{\left(1-\frac{n_{0}}{2}\right)\,s}\leq C_{0}\,s^{C_{0}}\,e^{-\frac{s}{2}},$$

which yields (2.4). If case (ii) occurs with $n_{0}=2$, by definition of $P_{2}$, we derive from (3.9) that there is a symmetric, real (ℓ×ℓ)-matrix ℬ such that

$$P_{2}\,g\,\left(y,s\right)=\frac{1}{s^{2}}\,\left(\frac{1}{2}\,{\bar{y}}^{T}\,ℬ\,\bar{y}-\mathrm{tr}\left(ℬ\right)\right)+o\,\left(\frac{1}{s^{2}}\right),$$

which is (2.3). This concludes the proof of Proposition 2.1.

### ${𝒞}^{1,\frac{1}{2}-\eta }$ -Regularity of the Blow-Up Set

3.2

We give the proof of Proposition 2.8 in this section. The proof uses the argument given in [17] treated for the case ℓ=1. Here we shall exploit the refined estimate (2.10) to obtain a geometric constraint on the blow-up set. Without loss of generality, we assume $\hat{a}=0$ and $Q_{\hat{a}}=\mathrm{Id}$. Under the hypotheses of Proposition 2.8, we know that $\gamma \in {𝒞}^{1}\,\left({\left(-\delta_{1},\delta_{1}\right)}^{N-\ell },{\mathbb{R}}^{\ell }\right)$ with ℓ∈{1,…,N-1}. If we introduce

$$\Gamma \,\left(\tilde{x}\right)=\left(\gamma_{1}\,\left(\tilde{x}\right),\ldots ,\gamma_{\ell }\,\left(\tilde{x}\right),\tilde{x}\right),\tilde{x}=\left(x_{\ell +1},\ldots ,x_{N}\right),$$

then

Im⁡Γ∩B⁢(0,2⁢δ)=graph⁡(γ)∩B⁢(0,2⁢δ)=Sδ.

Consider $\tilde{x}$ and $\tilde{h}$ in ${\mathbb{R}}^{N-\ell }$ such that $\tilde{x}$ as well as $\tilde{x}+\tilde{h}$ are in $B\,\left(0,\delta_{1}\right)$ and $\Gamma \,\left(\tilde{x}\right)$ as well as $\Gamma \,\left(\tilde{x}+\tilde{h}\right)$ are in $S_{\delta }$. For all $t\in \left[T-e^{-s_{0}-1},T\right)$ such that $\left|\Gamma \,\left(\tilde{x}\right)-\Gamma \,\left(\tilde{x}+\tilde{h}\right)\right|\leq \sqrt{\left(T-t\right)\,\left|\log\left(T-t\right)\right|}$, we use (2.10) with $x=a=\Gamma \,\left(\tilde{x}+\tilde{h}\right)$, then with $x=\Gamma \,\left(\tilde{x}+\tilde{h}\right)$ and $a=\Gamma \,\left(\tilde{x}\right)$ to find that

(3.11) $$\left\{ \begin{array}{cc} \left|{\left(T-t\right)}^{\frac{1}{p-1}}\,u\,\left(\Gamma \,\left(\tilde{x}+\tilde{h}\right),t\right)-w_{ℬ\,\left(\Gamma \,\left(\tilde{x}+\tilde{h}\right)\right)}\,\left(0,s\right)\right| & \leq C\,e^{-\frac{s}{2}}\,s^{\frac{3}{2}+C_{0}},\\ \left|{\left(T-t\right)}^{\frac{1}{p-1}}\,u\,\left(\Gamma \,\left(\tilde{x}+\tilde{h}\right),t\right)-w_{ℬ\,\left(\Gamma \,\left(\tilde{x}\right)\right)}\,\left({\bar{y}}_{\Gamma \,\left(\tilde{x}\right),\Gamma \,\left(\tilde{x}+\tilde{h}\right),s},s\right)\right| & \leq C\,e^{-\frac{s}{2}}\,s^{\frac{3}{2}+C_{0}}, \end{array}\right.$$

where ${\bar{y}}_{\Gamma \,\left(\tilde{x}\right),\Gamma \,\left(\tilde{x}+\tilde{h}\right),s}$ is defined as

(3.12) $${\bar{y}}_{a_{1},a_{2},s}=e^{\frac{s}{2}}\,\left(\left(a_{1}-a_{2}\right)\cdot Q_{a_{1}}\,e_{1},\ldots ,\left(a_{1}-a_{2}\right)\cdot Q_{a_{1}}\,e_{\ell }\right).$$

Since Γ is ${𝒞}^{1}$, we have

$$\left|\Gamma \,\left(\tilde{x}+\tilde{h}\right)-\Gamma \,\left(\tilde{x}\right)\right|\leq C\,\left|\tilde{h}\right|.$$

Let us fix $t=\tilde{t}\,\left(\tilde{x},\tilde{h}\right)$ such that

(3.13) $$\left|\Gamma \,\left(\tilde{x}+\tilde{h}\right)-\Gamma \,\left(\tilde{x}\right)\right|=\sqrt{\left(T-\tilde{t}\right)\,\left|\log\left(T-\tilde{t}\right)\right|},$$

and take $\tilde{h}\in B_{N-\ell }\,\left(0,h_{1}\,\left(s_{0}\right)\right)$ for some $h_{1}\,\left(s_{0}\right)>0$. Then we have $\tilde{t}\geq T-e^{-s_{0}-1}$. Hence, if $\tilde{s}=-\log\left(T-\tilde{t}\right)$, by (3.11) we have

(3.14) $$\left|w_{ℬ\,\left(\Gamma \,\left(\tilde{x}+\tilde{h}\right)\right)}\,\left(0,\tilde{s}\right)-w_{ℬ\,\left(\Gamma \,\left(\tilde{x}\right)\right)}\,\left({\bar{y}}_{\Gamma \,\left(\tilde{x}\right),\Gamma \,\left(\tilde{x}+\tilde{h}\right),\tilde{s}},\tilde{s}\right)\right|\leq C\,e^{-\frac{\tilde{s}}{2}}\,{\tilde{s}}^{\frac{3}{2}+C_{0}}.$$

Similarly, by changing the roles of $\tilde{x}$ and $\tilde{x}+\tilde{h}$, we get

(3.15) $$\left|w_{ℬ\,\left(\Gamma \,\left(\tilde{x}\right)\right)}\,\left(0,\tilde{s}\right)-w_{ℬ\,\left(\Gamma \,\left(\tilde{x}+\tilde{h}\right)\right)}\,\left({\bar{y}}_{\Gamma \,\left(\tilde{x}+\tilde{h}\right),\Gamma \,\left(\tilde{x}\right),\tilde{s}},\tilde{s}\right)\right|\leq C\,e^{-\frac{\tilde{s}}{2}}\,{\tilde{s}}^{\frac{3}{2}+C_{0}},$$

where ${\bar{y}}_{\Gamma \,\left(\tilde{x}+\tilde{h}\right),\Gamma \,\left(\tilde{x}\right),\tilde{s}}$ is defined as in (3.12).

From a Taylor expansion for $w_{ℬ}\,\left(\bar{y},\tilde{s}\right)$ near $\bar{y}=0$, we write

(3.16) $$w_{ℬ}\,\left(\bar{y},\tilde{s}\right)=w_{ℬ}\,\left(0,\tilde{s}\right)+\bar{y}\cdot \nabla w_{ℬ}\,\left(0,\tilde{s}\right)+\frac{1}{2}\,{\bar{y}}^{T}\,\nabla^{2}w_{ℬ}\,\left(0,\tilde{s}\right)\,\bar{y}+𝒪\,\left({\left|\bar{y}\right|}^{3}\,\left|\nabla^{3}w_{ℬ}\,\left(z,\tilde{s}\right)\right|\right),$$

for some *z* between 0 and $\bar{y}$.

Since (2.2) and (2.7) also hold in ${𝒞}_{\mathrm{loc}}^{k}$ by parabolic regularity, we deduce that

$$\left|\nabla w_{ℬ}\,\left(0,\tilde{s}\right)\right|=𝒪\,\left(\frac{\log\tilde{s}}{{\tilde{s}}^{2}}\right),\nabla^{2}w_{ℬ}\,\left(0,\tilde{s}\right)=-\frac{\kappa }{4\,p\,\tilde{s}}\,I_{\ell \times \ell }+𝒪\,\left(\frac{\log\tilde{s}}{{\tilde{s}}^{2}}\right).$$

From [11, Theorem 1], we know that

$${∥\nabla^{3}w_{ℬ}\,\left(\tilde{s}\right)∥}_{L^{\infty }}\leq \frac{C_{3}}{{\tilde{s}}^{\frac{3}{2}}}.$$

Substituting all these above estimates into (3.16) yields

$$w_{ℬ}\,\left(\bar{y},\tilde{s}\right)\leq w_{ℬ}\,\left(0,\tilde{s}\right)-\frac{\kappa }{8\,p\,\tilde{s}}\,{\left|\bar{y}\right|}^{2}+\frac{C_{3}\,{\left|\bar{y}\right|}^{3}}{6\,{\tilde{s}}^{\frac{3}{2}}}+\frac{C\,\log\tilde{s}}{{\tilde{s}}^{2}}.$$

Therefore, we have

(3.17) $$w_{ℬ}\,\left(\bar{y},\tilde{s}\right)\leq w_{ℬ}\,\left(0,\tilde{s}\right)-\frac{\kappa }{16\,p\,\tilde{s}}\,{\left|\bar{y}\right|}^{2}\;\text{for all }\,\left|\bar{y}\right|\leq \frac{3\,\kappa }{8\,C_{3}\,p}\,\sqrt{\tilde{s}}.$$

We claim from (3.14), (3.15) and (3.17) the following:

(3.18) $$\left|w_{ℬ\,\left(\Gamma \,\left(\tilde{x}\right)\right)}\,\left(0,\tilde{s}\right)-w_{ℬ\,\left(\Gamma \,\left(\tilde{x}+\tilde{h}\right)\right)}\,\left(0,\tilde{s}\right)\right|\leq C\,e^{-\frac{\tilde{s}}{2}}\,{\tilde{s}}^{\frac{3}{2}+C_{0}}.$$

Indeed, if $w_{ℬ\,\left(\Gamma \,\left(\tilde{x}\right)\right)}\,\left(0,\tilde{s}\right)-w_{ℬ\,\left(\Gamma \,\left(\tilde{x}+\tilde{h}\right)\right)}\,\left(0,\tilde{s}\right)\geq 0$, then by (3.17) and (3.15) we have

$$0\leq w_{ℬ\,\left(\Gamma \,\left(\tilde{x}\right)\right)}\,\left(0,\tilde{s}\right)-w_{ℬ\,\left(\Gamma \,\left(\tilde{x}+\tilde{h}\right)\right)}\,\left(0,\tilde{s}\right)$$

$$\leq w_{ℬ\,\left(\Gamma \,\left(\tilde{x}\right)\right)}\,\left(0,\tilde{s}\right)-w_{ℬ\,\left(\Gamma \,\left(\tilde{x}+\tilde{h}\right)\right)}\,\left({\bar{y}}_{\Gamma \,\left(\tilde{x}+\tilde{h}\right),\Gamma \,\left(\tilde{x}\right),\tilde{s}},\tilde{s}\right)$$

$$\leq C\,e^{-\frac{\tilde{s}}{2}}\,{\tilde{s}}^{\frac{3}{2}+C_{0}}.$$

If $w_{ℬ\,\left(\Gamma \,\left(\tilde{x}\right)\right)}\,\left(0,\tilde{s}\right)-w_{ℬ\,\left(\Gamma \,\left(\tilde{x}+\tilde{h}\right)\right)}\,\left(0,\tilde{s}\right)\leq 0$, then we do as above and use (3.14) instead of (3.15) to obtain (3.18).

From (3.18), (3.14) and (3.17), we get

$$\frac{\kappa }{16\,p\,\tilde{s}}\,{\left|{\bar{y}}_{\Gamma \,\left(\tilde{x}\right),\Gamma \,\left(\tilde{x}+\tilde{h}\right),\tilde{s}}\right|}^{2}\leq w_{ℬ\,\left(\Gamma \,\left(\tilde{x}\right)\right)}\,\left(0,\tilde{s}\right)-w_{ℬ\,\left(\Gamma \,\left(\tilde{x}\right)\right)}\,\left({\bar{y}}_{\Gamma \,\left(\tilde{x}\right),\Gamma \,\left(\tilde{x}+\tilde{h}\right),\tilde{s}},\tilde{s}\right)\leq C\,e^{-\frac{\tilde{s}}{2}}\,{\tilde{s}}^{\frac{3}{2}+C_{0}}.$$

Hence, we obtain

(3.19) $${\left|{\bar{y}}_{\Gamma \,\left(\tilde{x}\right),\Gamma \,\left(\tilde{x}+\tilde{h}\right),\tilde{s}}\right|}^{2}\leq C\,e^{-\frac{\tilde{s}}{2}}\,{\tilde{s}}^{\frac{5}{2}+C_{0}}.$$

From the definition (3.12), we have

(3.20) $$\left|{\bar{y}}_{\Gamma \,\left(\tilde{x}\right),\Gamma \,\left(\tilde{x}+\tilde{h}\right),\tilde{s}}\right|=e^{\frac{\tilde{s}}{2}}\,d\,\left(\Gamma \,\left(\tilde{x}\right),\pi_{\Gamma \,\left(\tilde{x}+\tilde{h}\right)}\right),$$

where we recall $\pi_{\Gamma \,\left(\tilde{x}+\tilde{h}\right)}$ is the tangent plan of *S* at $\Gamma \,\left(\tilde{x}+\tilde{h}\right)$. On the other hand, we claim that

(3.21) $$d\,\left(\Gamma \,\left(\tilde{x}\right),T_{\Gamma \,\left(\tilde{x}+\tilde{h}\right)}\right)\geq \frac{\left|\gamma_{i}\,\left(\tilde{x}+\tilde{h}\right)-\gamma_{i}\,\left(\tilde{x}\right)-\tilde{h}\cdot \nabla \gamma_{i}\,\left(\tilde{x}\right)\right|}{\sqrt{1+{\left|\nabla \gamma_{i}\,\left(\tilde{x}\right)\right|}^{2}}},$$

where $S_{i}$ is the surface of equation $x_{i}=\gamma_{i}\,\left(\tilde{x}\right)$, and $T_{i,\Gamma \,\left(\tilde{x}+\tilde{h}\right)}$ is the tangent plan of $S_{i}$ at $\Gamma \,\left(\tilde{x}+\tilde{h}\right)$. Indeed, we note that

$$d\,\left(\Gamma \,\left(\tilde{x}\right),T_{i,\Gamma \,\left(\tilde{x}+\tilde{h}\right)}\right)=\frac{\left|\gamma_{i}\,\left(\tilde{x}+\tilde{h}\right)-\gamma_{i}\,\left(\tilde{x}\right)-\tilde{h}\cdot \nabla \gamma_{i}\,\left(\tilde{x}\right)\right|}{\sqrt{1+{\left|\nabla \gamma_{i}\,\left(\tilde{x}\right)\right|}^{2}}},$$

and Im⁡Γ⊂Si, hence, (3.21) follows from $d\,\left(\Gamma \,\left(\tilde{x}\right),T_{\Gamma \,\left(\tilde{x}+\tilde{h}\right)}\right)\geq d\,\left(\Gamma \,\left(\tilde{x}\right),T_{i,\Gamma \,\left(\tilde{x}+\tilde{h}\right)}\right)$.

Combining (3.19), (3.20), (3.21) together with the relation $\tilde{s}=-\log\left(T-\tilde{t}\right)$ yields

$${\left|\gamma_{i}\,\left(\tilde{x}+\tilde{h}\right)-\gamma_{i}\,\left(\tilde{x}\right)-\tilde{h}\cdot \nabla \gamma_{i}\,\left(\tilde{x}\right)\right|}^{2}\leq C\,{\left(T-\tilde{t}\right)}^{\frac{3}{2}}\,{\left|\log\left(T-\tilde{t}\right)\right|}^{\frac{5}{2}+C_{0}}.$$

If we denote $A=\left|\Gamma \,\left(\tilde{x}+\tilde{h}\right)-\Gamma \,\left(\tilde{x}\right)\right|\leq C\,\left|\tilde{h}\right|$, then by relation (3.13) we have

$$\left|\log\left(T-\tilde{t}\right)\right|∼2\,\left|\log A\right|,T-\tilde{t}∼\frac{A^{2}}{2\,\left|\log A\right|}\;\text{as }\,A\to 0.$$

Hence,

$${\left|\gamma_{i}\,\left(\tilde{x}+\tilde{h}\right)-\gamma_{i}\,\left(\tilde{x}\right)-\tilde{h}\cdot \nabla \gamma_{i}\,\left(\tilde{x}\right)\right|}^{2}\leq C\,A^{3}\,{\left|\log A\right|}^{1+C_{0}}\leq C\,{\left|\tilde{h}\right|}^{3}\,{\left|\log\left|\tilde{h}\right|\right|}^{1+C_{0}},$$

which yields (2.23). This concludes the proof of Proposition 2.8.

### 3.3 A Geometric Constraint Linking the Blow-Up Behavior of the Solution to the Regularity of the Blow-Up Set

This section is devoted to the proof of Proposition 2.7. The proof follows ideas given in [20]. By the hypothesis, we have $\gamma_{a}\in {𝒞}^{1,\alpha^{*}}\,\left({\left(-\epsilon_{a},\epsilon_{a}\right)}^{N-\ell },{\mathbb{R}}^{\ell }\right)$ for some $\alpha^{*}\in \left(0,\frac{1}{2}\right)$ and $\epsilon_{a}>0$, and $\gamma_{a,i}\,\left(0\right)=\nabla \gamma_{a,i}\,\left(0\right)=0$. Thus, for all $\left|\tilde{\xi }\right|<\epsilon_{a}$,

(3.22) $$\left|\gamma_{a,i}\,\left(\tilde{\xi }\right)\right|\leq C\,{\left|\tilde{\xi }\right|}^{1+\alpha^{*}}\;\text{and}\;\left|\nabla \gamma_{a,i}\,\left(\tilde{\xi }\right)\right|\leq C\,{\left|\tilde{\xi }\right|}^{\alpha^{*}}.$$

In what follows, k∈{ℓ+1,…,N} is fixed, and we use indexes *i* and *m* for the range 1,…,ℓ, index *j* for the range ℓ+1,…,N.

We now use (3.22) to approximate all the terms appearing in (2.20).

(a) Term $\tau_{k}\,\left(a\right)\cdot \eta_{i}\,\left(b\right)$. From the local coordinates (2.21), we have

$$\eta_{i}\,\left(b\right)=\frac{1}{\sqrt{1+{\left|\nabla \gamma_{a,i}\,\left(e^{-\frac{s}{2}}\,\tilde{y}\right)\right|}^{2}}}\,\left(\eta_{i}\,\left(a\right)-\sum_{j=\ell +1}^{N}\frac{\partial \gamma_{a,i}}{\partial \xi_{j}}\,\left(e^{-\frac{s}{2}}\,\tilde{y}\right)\,\tau_{j}\,\left(a\right)\right).$$

Using (3.22) and the fact that $\tau_{k}\,\left(a\right)\cdot \eta_{i}\,\left(a\right)=0$ and $\tau_{k}\,\left(a\right)\cdot \tau_{j}\,\left(a\right)=\delta_{k,j}$, we obtain

$$\left|\tau_{k}\,\left(a\right)\cdot \eta_{i}\,\left(b\right)+\frac{\partial \gamma_{a,i}}{\partial \xi_{k}}\,\left(e^{-\frac{s}{2}}\,\tilde{y}\right)\right|=\left|\left(1-\frac{1}{\sqrt{1+{\left|\nabla \gamma_{a,i}\,\left(e^{-\frac{s}{2}}\,\tilde{y}\right)\right|}^{2}}}\right)\,\frac{\partial \gamma_{a,i}}{\partial \xi_{k}}\,\left(e^{-\frac{s}{2}}\,\tilde{y}\right)\right|$$

$$\leq \left|\frac{\partial \gamma_{a,i}}{\partial \xi_{k}}\,\left(e^{-\frac{s}{2}}\,\tilde{y}\right)\right|\,{\left|\nabla \gamma_{a,i}\,\left(e^{-\frac{s}{2}}\,\tilde{y}\right)\right|}^{2}$$

(3.23) $$\leq \left|\frac{\partial \gamma_{a,i}}{\partial \xi_{k}}\,\left(e^{-\frac{s}{2}}\,\tilde{y}\right)\right|\,e^{-\alpha^{*}\,s}.$$

(b) Term $\tau_{k}\,\left(a\right)\cdot \tau_{j}\,\left(b\right)$. From (2.21) and (3.22), we have

$$\left|b-a\right|\leq \left|\sum_{i=1}^{\ell }\gamma_{a,i}\,\left(e^{-\frac{s}{2}}\,\tilde{y}\right)\right|+e^{-\frac{s}{2}}\,\left|\tilde{y}\right|\leq C\,e^{-\frac{s}{2}}.$$

Since $\eta_{i}$ and $\tau_{j}$ are ${𝒞}^{\alpha^{*}}$, it holds that

$$\left|\eta_{i}\,\left(a\right)-\eta_{i}\,\left(b\right)\right|+\left|\tau_{j}\,\left(a\right)-\tau_{j}\,\left(b\right)\right|\leq C\,{\left|a-b\right|}^{\alpha^{*}}\leq C\,e^{-\alpha^{*}\,\frac{s}{2}}.$$

It follows that

(3.24) $$\left\{ \begin{array}{cc} \left|\eta_{i}\,\left(a\right)\cdot \eta_{m}\,\left(b\right)-\delta_{i,m}\right|+\left|\tau_{k}\,\left(a\right)\cdot \tau_{j}\,\left(b\right)-\delta_{k,j}\right| & \leq C\,e^{-\alpha^{*}\,\frac{s}{2}},\\ \left|\eta_{i}\,\left(a\right)\cdot \tau_{j}\,\left(b\right)\right|+\left|\eta_{i}\,\left(b\right)\cdot \tau_{j}\,\left(a\right)\right| & \leq C\,e^{-\alpha^{*}\,\frac{s}{2}}. \end{array}\right.$$

(c) The point Y⁢(a,y,s). Using (2.16), (2.19) and (2.21), we write

$$Y_{m}=Y\cdot e_{m}=\left(Q_{a}\,y+e^{\frac{s}{2}}\,\left(a-b\right)\right)\cdot Q_{b}\,e_{m}$$

$$=\left\{\sum_{i=1}^{\ell }y_{i}\,\eta_{i}\,\left(a\right)+\sum_{j=\ell +1}^{N}y_{j}\,\tau_{j}\,\left(a\right)-e^{\frac{s}{2}}\,\left[\sum_{i=1}^{\ell }\gamma_{a,i}\,\left(e^{-\frac{s}{2}}\,\tilde{y}\right)\,\eta_{i}\,\left(a\right)+\sum_{j=\ell +1}^{N}e^{-\frac{s}{2}}\,y_{j}\,\tau_{j}\,\left(a\right)\right]\right\}\cdot Q_{b}\,e_{m}$$

$$=\left\{\sum_{i=1}^{\ell }\left[y_{i}-e^{\frac{s}{2}}\,\gamma_{a,i}\,\left(e^{-\frac{s}{2}}\,\tilde{y}\right)\right]\,\eta_{i}\,\left(a\right)\right\}\cdot Q_{b}\,e_{m}.$$

From (2.16), we write for m∈{1,…,ℓ},

$$Y_{m}-y_{m}=\left\{\left(y_{m}-e^{\frac{s}{2}}\,\gamma_{a,m}\,\left(e^{-\frac{s}{2}}\,\tilde{y}\right)\right)\,\eta_{m}\,\left(a\right)\cdot \eta_{m}\,\left(b\right)-y_{m}\,\eta_{m}\,\left(a\right)\cdot \eta_{m}\,\left(a\right)\right\}$$

$$+\sum_{i=1,i\neq m}^{\ell }\left(y_{i}-e^{\frac{s}{2}}\,\gamma_{a,i}\,\left(e^{-\frac{s}{2}}\,\tilde{y}\right)\right)\,\eta_{i}\,\left(a\right)\cdot \eta_{m}\,\left(b\right),$$

and for n∈{ℓ+1,…,N},

$$Y_{n}=\sum_{i=1}^{\ell }\left(y_{i}-e^{\frac{s}{2}}\,\gamma_{a,i}\,\left(e^{-\frac{s}{2}}\,\tilde{y}\right)\right)\,\eta_{i}\,\left(a\right)\cdot \tau_{n}\,\left(b\right).$$

Using (3.24) yields

$$\left|Y_{m}-y_{m}\right|\leq C\,e^{-\alpha^{*}\,\frac{s}{2}}\;\text{and}\;\left|Y_{k}\right|\leq C\,e^{-\alpha^{*}\,\frac{s}{2}}.$$

Hence, if we write

$$\bar{Y}=\left(Y_{1},\ldots ,Y_{\ell }\right)\;\text{and}\;\tilde{Y}=\left(Y_{\ell +1},\ldots ,Y_{N}\right),$$

then

(3.25) $$\left|\bar{y}-\bar{Y}\right|\leq C\,e^{-\alpha^{*}\,\frac{s}{2}}\;\text{and}\;\left|\tilde{Y}\right|\leq C\,e^{-\alpha^{*}\,\frac{s}{2}}.$$

(d) Term $\frac{\partial w_{b}}{\partial y_{i}}\,\left(Y,s\right)$. From Proposition 2.5 and the parabolic regularity, we have that

(3.26) $$\underset{s\geq s^{\prime }}{\sup}{∥w_{b}\,\left(y,s\right)-w_{ℬ\,\left(b\right)}\,\left(\bar{y},s\right)∥}_{W_{\mathrm{loc}}^{2,\infty }\left(|\bar{y}|<2\right)}\leq C\,e^{-\frac{s}{2}}\,s^{C_{0}}.$$

This implies

(3.27) $$\left|\frac{\partial w_{b}}{\partial y_{i}}\,\left(Y,s\right)-\frac{\partial w_{ℬ\,\left(b\right)}}{\partial y_{i}}\,\left(\bar{y},s\right)\right|+\sum_{m=\ell +1}^{N}\left|\frac{\partial w_{b}}{\partial y_{m}}\,\left(Y,s\right)\right|+\underset{\left|z\right|<2,\left(m,n\right)\neq \left(i,i\right),i\geq \ell +1}{\sup}\left|\frac{\partial^{2}w_{b}}{\partial y_{m}\,\partial y_{n}}\,\left(z,s\right)\right|\leq C\,e^{-\frac{s}{2}}\,s^{C_{0}}.$$

Similarly, from (2.1) and (2.18),

(3.28) $$\underset{s\geq -\log T}{\sup}{∥w_{a}\,\left(y,s\right)-\left\{\kappa +\frac{\kappa }{2\,p\,s}\,\left(\ell -\frac{{\left|\bar{y}\right|}^{2}}{2}\right)\right\}∥}_{W_{\mathrm{loc}}^{2,\infty }\left(|\bar{y}|<2\right)}\leq C\,\frac{\log s}{s^{2}}.$$

From (3.26) and (3.28), we deduce that

(3.29) $$\underset{s\geq s^{\prime \prime }}{\sup}{∥w_{ℬ\,\left(a\right)}\,\left(y,s\right)-\left\{\kappa +\frac{\kappa }{2\,p\,s}\,\left(\ell -\frac{{\left|\bar{y}\right|}^{2}}{2}\right)\right\}∥}_{W_{\mathrm{loc}}^{2,\infty }\left(|\bar{y}|<2\right)}\leq C\,\frac{\log s}{s^{2}}.$$

Using (3.29), we have for |z|≤2,

$$\left|\frac{\partial^{2}w_{ℬ\,\left(b\right)}}{\partial y_{i}^{2}}\,\left(z,s\right)+\frac{\kappa }{2\,p\,s}\right|\leq C\,\frac{\log s}{s^{2}}\;\text{and}\;\left|\frac{\partial^{2}w_{ℬ\,\left(b\right)}}{\partial y_{i}\,\partial y_{m}}\,\left(z,s\right)\right|\leq C\,\frac{\log s}{s^{2}},m\neq i.$$

Note that $\partial w_{ℬ\,\left(b\right)}/\partial y_{i}\,\left(0,s\right)=0$. We then take the Taylor expansion of $\partial w_{ℬ\,\left(b\right)}/\partial y_{i}\,\left(\bar{y},s\right)$ near $\bar{y}=0$ up to the first order to get

$$\left|\frac{\partial w_{ℬ\,\left(b\right)}}{\partial y_{i}}\,\left(\bar{y},s\right)+Y_{i}\,\frac{\kappa }{2\,p\,s}\right|\leq C\,\left|\bar{y}\right|\,\frac{\log s}{s^{2}}.$$

Using (3.27) and (3.25) yields

(3.30) $$\left|\frac{\partial w_{b}}{\partial y_{i}}\,\left(Y,s\right)+y_{i}\,\frac{\kappa }{2\,p\,s}\right|\leq C\,e^{-\frac{s}{2}}\,s^{C_{0}}+C\,\left|\bar{y}\right|\,\frac{\log s}{s^{2}}+\frac{C}{s}\,e^{-\alpha^{*}\,\frac{s}{2}}.$$

(e) Term $\frac{\partial w_{b}}{\partial y_{j}}\,\left(Y,s\right)$. We just use (3.27) and (3.25) to get

(3.31) $$\left|\frac{\partial w_{b}}{\partial y_{j}}\,\left(Y,s\right)-\frac{\partial w_{b}}{\partial y_{j}}\,\left(\bar{y},0,\ldots ,0,s\right)\right|\leq C\,e^{-\left(1+\alpha^{*}\right)\,\frac{s}{2}}.$$

Estimate (2.22) then follows by substituting (3.31), (3.30), (3.27), (3.23) and (3.24) into (2.20). This concludes the proof of Proposition 2.7.

### 3.4 Further Refined Asymptotic Behavior

We prove Proposition 2.10 in this subsection. We first refine estimate (2.8) and find the following terms in the expansion which is of order $e^{-\frac{s}{2}}$. Using the geometric constraint, we show that all terms of order $e^{-\frac{s}{2}}$ must be identically zero, which gives a better estimate for ${∥W_{a}\,\left(Q_{a}\,y,s\right)-w_{ℬ\,\left(a\right)}\,\left(\bar{y},s\right)∥}_{L_{\rho }^{2}}$. We then repeat the process and use again Proposition 2.7 in order to get the term of order $\frac{1}{s}\,e^{-\frac{s}{2}}$ and conclude the proof of Proposition 2.10.

Let us define

(3.32) $$g_{a}\,\left(y,s\right)=W_{a}\,\left(Q_{a}\,y,s\right)-w_{ℬ\,\left(a\right)}\,\left(\bar{y},s\right)$$

and

$$I_{a}{\left(s\right)}^{2}=∥g_{a}\left(s\right){∥}_{L_{\rho }^{2}}^{2},l_{a,n}\left(s\right)=∥P_{n}g_{a}\left(s\right){∥}_{L_{\rho }^{2}},r_{a,k}\left(s\right)=∥\sum_{n\geq k}P_{n}g_{a}\left(s\right)∥{}_{L_{\rho }^{2}}.$$

From (2.8), we have

(3.33) $$I_{a}\,\left(s\right)=𝒪\,\left(e^{-\frac{s}{2}}\,s^{\mu }\right)\;\text{for some }\,\mu >0.$$

Note that Lemma 3.2 also holds with $W_{1}=W_{a}$ and $W_{2}=w_{ℬ}$. We claim the following:

Lemma 3.4 *Assume that $I_{a}\,\left(s\right)=O\,\left(e^{-\frac{s}{2}}\,s^{\mu_{0}}\right)$ for some $\mu_{0}\in R$. There exists $s_{4}>0$ such that for all $s\geq s_{4}$,*

(3.34) $$\sum_{n=0}^{2}l_{a,n}\,\left(s\right)+r_{a,4}\,\left(s\right)\leq C\,e^{-\frac{s}{2}}\,s^{\mu_{0}-1}$$

*and*

(3.35) $$\left|\frac{d}{d\,s}\,\left(g_{a,\beta }\,\left(s\right)\,e^{\frac{s}{2}}\,s^{\left|\bar{\beta }\right|}\right)\right|\leq C\,s^{\left|\bar{\beta }\right|+\mu_{0}-\frac{3}{2}}\;\text{for all }\,\beta \in {\mathbb{N}}^{N},\left|\beta \right|=3,$$

*where $\bar{\beta }=\left(\beta_{1},\ldots ,\beta_{\ell }\right)$, $\left|\bar{\beta }\right|=\sum_{i=1}^{\ell }\beta_{i}$.*

**Proof.**

By (3.4) and (3.7), we can write for all $s\geq s_{3}$,

$$\left|\frac{d}{d\,s}\,\left(l_{a,n}\,\left(s\right)\,e^{\left(n/2-1\right)\,s}\right)\right|\leq C\,e^{\left(n/2-\frac{3}{2}\right)\,s}\,s^{\mu_{0}-1},n=0,1,2,$$

and

$$\left|\frac{d}{d\,s}\,\left(r_{a,4}\,\left(s\right)\,e^{s}\right)\right|\leq C\,e^{\frac{s}{2}}\,s^{\mu_{0}-1}.$$

Estimate (3.34) then follows after integration of the above inequalities. As for (3.35), we just use (3.6) and (3.34) (note that $l_{a,5}\leq r_{a,4}$ by definition (3.3)). ∎

Using (3.33) and applying Lemma 3.4 a finite number of steps, we obtain the following:

Lemma 3.5 *There exist $s_{5}>0$ and continuous functions $a\to \lambda_{\beta }\,\left(a\right)$ for all $\beta \in N^{N}$ with |β|=3 and $\left|\bar{\beta }\right|=\sum_{i=1}^{\ell }\beta_{i}=0$ such that for all $a\in S_{\delta }$ and $s\geq s_{5}$,*

$${∥g_{a}\,\left(y,s\right)-e^{-\frac{s}{2}}\,\sum_{\left|\beta \right|=3,\left|\bar{\beta }\right|=0}\lambda_{\beta }\,\left(a\right)\,h_{\beta }\,\left(y\right)∥}_{L_{\rho }^{2}}\leq C\,e^{-\frac{s}{2}}\,s^{d-\frac{1}{2}},$$

*for some $d\in \left(0,\frac{1}{2}\right)$, where $h_{\beta }$ is defined by (3.2).*

**Proof.**

We first show that there is $s_{5}>0$ such that

(3.36) $$I_{a}\,\left(s\right)\leq C\,e^{-\frac{s}{2}}\,s^{d}\;\text{for some }\,d\in \left(0,\frac{1}{2}\right)\;\text{for all }\,s\geq s_{5}.$$

From (3.33), if $\mu \in \left(0,\frac{1}{2}\right)$, we are done. If $\mu \geq \frac{1}{2}$, we apply Lemma 3.4 with $\mu_{0}=\mu$ to get

$$\sum_{n=0}^{2}l_{a,n}\,\left(s\right)+r_{a,4}\,\left(s\right)\leq C\,e^{-\frac{s}{2}}\,s^{\mu -1}$$

and

$$\left|g_{a,\beta }\,\left(s\right)\right|\leq C\,e^{-\frac{s}{2}}\,s^{\mu -\frac{1}{2}}\;\text{for all }\,\left|\beta \right|=3.$$

Hence,

$$I_{a}\,\left(s\right)\leq C\,e^{-\frac{s}{2}}\,s^{\mu -\frac{1}{2}}.$$

Estimate (3.36) then follows by repeating this process a finite number of steps.

Now using (3.36) and Lemma 3.4 with $\mu_{0}=d$, we distinguish the following two cases:

• If |β|=3 and $\left|\bar{\beta }\right|\geq 1$, we integrate (3.35) on [s,+∞) to derive
$$\left|g_{a,\beta }\,\left(s\right)\right|\leq C\,e^{-\frac{s}{2}}\,s^{d-\frac{1}{2}}\;\text{for all }\,\left|\beta \right|=3,\left|\bar{\beta }\right|\geq 1.$$
• If |β|=3 and $\left|\bar{\beta }\right|=0$, by integrating (3.35) on $\left[s_{5},s\right]$, we deduce that there exist continuous functions $a\to \lambda_{\beta }\,\left(a\right)$ such that
$$\left|g_{a,\beta }\,\left(s\right)-\lambda_{\beta }\,\left(a\right)\,e^{-\frac{s}{2}}\right|\leq C\,e^{-\frac{s}{2}}\,s^{d-\frac{1}{2}}\;\text{for all }\,\left|\beta \right|=3,\left|\bar{\beta }\right|=0.$$

This concludes the proof of Lemma 3.5. ∎

Now we shall use the geometric constraint on the asymptotic behavior of the solution given in Proposition 2.7 to show that all the coefficients $\lambda_{\beta }\,\left(a\right)$ with |β|=3 and $\bar{\beta }=0$ in Lemma 3.5 have to be identically zero. In particular, we claim the following:

Lemma 3.6 *There exists $s_{6}>0$ such that for all $s\geq s_{6}$,*

$${∥g_{a}\,\left(s\right)∥}_{L_{\rho }^{2}}\leq C\,e^{-\frac{s}{2}}\,s^{d-\frac{1}{2}}\;\text{for some }\,d\in \left(0,\frac{1}{2}\right)\,\text{ and all }\,a\in S_{\delta }.$$

**Proof.**

Consider $a\in S_{\delta }$. We aim at proving that

$$\lambda_{\beta }\,\left(a\right)=0\;\text{for all }\,\beta \in {\mathbb{N}}^{N},\left|\beta \right|=3,\left|\bar{\beta }\right|=0,$$

where $\lambda_{\beta }\,\left(a\right)$ is introduced in Lemma 3.5 and $\left|\bar{\beta }\right|=\sum_{i=1}^{\ell }\beta_{i}$.

From (2.18), (3.32) and the fact that the estimate given in Lemma 3.5 also holds in $W^{2,\infty }\left(|y|<2\right)$ by parabolic regularity, we write for all k≥ℓ+1 and $s\geq s_{5}+1$,

(3.37) $$\underset{a\in S_{\delta },\left|y\right|<2}{\sup}\left|\frac{\partial w_{a}}{\partial y_{k}}\,\left(y,s\right)-e^{-\frac{s}{2}}\,\sum_{\left|\beta \right|=3,\bar{\beta }=0}\lambda_{\beta }\,\left(a\right)\,\frac{\partial h_{\beta }}{\partial y_{k}}\,\left(y\right)\right|\leq C\,e^{-\frac{s}{2}}\,s^{d-\frac{1}{2}}.$$

Take $y=\left(\bar{y},\tilde{y}\right)$, where $\bar{y}=\left(y_{1},\ldots ,y_{\ell }\right)=\left(0,\ldots ,0\right)$ and $\tilde{y}\in B_{N-\ell }\,\left(0,1\right)$. Then we use Proposition 2.9 and (2.22) to obtain

(3.38) $$\left|\frac{\partial w_{a}}{\partial y_{k}}\,\left(y,s\right)-\frac{\partial w_{b}}{\partial y_{k}}\,\left(0,s\right)\right|\leq C\,e^{-\left(1+\alpha^{*}\right)\,\frac{s}{2}}\,s^{C_{0}}+C\,e^{-s}\,s^{C_{0}+1},$$

for some $\alpha^{*}\in \left(0,\frac{1}{2}\right)$.

From (3.37) and (3.38), we get

(3.39) $$\left|\sum_{\left|\beta \right|=3,\left|\bar{\beta }\right|=0}\lambda_{\beta }\,\left(a\right)\,\frac{\partial h_{\beta }}{\partial y_{k}}\,\left(y\right)-\sum_{\left|\beta \right|=3,\left|\bar{\beta }\right|=0}\lambda_{\beta }\,\left(b\right)\,\frac{\partial h_{\beta }}{\partial y_{k}}\,\left(0\right)\right|\leq C\,s^{d-\frac{1}{2}}.$$

From (2.21) and Proposition 2.9, we see that b→a as s→+∞. Since $a\to \lambda_{\beta }\,\left(a\right)$ is continuous, $d\in \left(0,\frac{1}{2}\right)$, $h_{\beta_{1}}\,\left(0\right)=\cdots =h_{\beta_{\ell }}\,\left(0\right)=h_{0}\,\left(0\right)=1$ from definition (3.2), and

$$\frac{\partial h_{\beta }}{\partial y_{k}}\,\left(y\right)=\beta_{k}\,h_{\beta_{k}-1}\,\left(y_{k}\right)\,\prod_{j=1,j\neq k}^{N}h_{\beta_{j}}\,\left(y_{j}\right),$$

we derive, by passing to the limit in (3.39),

$$\sum_{\left|\beta \right|=3,\left|\bar{\beta }\right|=0}\lambda_{\beta }\,\left(a\right)\,\beta_{k}\,h_{\beta_{k}-1}\,\left(y_{k}\right)\,\prod_{j=\ell +1,j\neq k}^{N}h_{\beta_{j}}\,\left(y_{j}\right)=\sum_{\left|\beta \right|=3,\left|\bar{\beta }\right|=0}\lambda_{\beta }\,\left(a\right)\,\beta_{k}\,h_{\beta_{k}-1}\,\left(0\right)\,\prod_{j=\ell +1,j\neq k}^{N}h_{\beta_{j}}\,\left(0\right).$$

By the orthogonality of the polynomials $h_{i}$, this yields

$$\beta_{k}\,\lambda_{\beta }\,\left(a\right)=0\;\text{for all }\,k\geq \ell +1\,\text{ and }\,\left|\beta \right|=3\,\text{ with }\,\left|\bar{\beta }\right|=0.$$

Take β arbitrary with |β|=3 and $\left|\bar{\beta }\right|=0$, then there exists k≥ℓ+1 such that $\beta_{k}\geq 1$, which implies that $\lambda_{\beta }\,\left(a\right)=0$. This ends the proof of Lemma 3.6. ∎

**Proof of Proposition 2.10.**

From Lemmas 3.6 and 3.4, we see that for all $s\geq s_{7}=\max\left\{s_{4},s_{5},s_{6}\right\}$,

$$\sum_{n=0}^{2}l_{a,n}\,\left(s\right)+r_{a,4}\,\left(s\right)\leq C\,s^{-\frac{s}{2}}\,s^{d-\frac{3}{2}}$$

and

(3.40) $$\left|\frac{d}{d\,s}\,\left(g_{a,\beta }\,\left(s\right)\,s^{\frac{s}{2}}\,s^{\left|\bar{\beta }\right|}\right)\right|\leq C\,e^{\left|\bar{\beta }\right|+d-2}\;\text{for all }\,\left|\beta \right|=3,$$

for some $d\in \left(0,\frac{1}{2}\right)$. Integrating (3.40) between *s* and +∞ if $\left|\bar{\beta }\right|=0$ and between $s_{7}$ and *s* if $\left|\bar{\beta }\right|\geq 1$, we get

$$\left|g_{a,\beta }\,\left(s\right)\right|\leq C\,e^{-\frac{s}{2}}\,s^{d-1}\;\text{for all }\,\left|\beta \right|=3.$$

Hence,

$$I_{a}\,\left(s\right)={∥g_{a}\,\left(s\right)∥}_{L_{\rho }^{2}}\leq C\,e^{-\frac{s}{2}}\,s^{d-1}\;\text{for all }\,s\geq s_{7}.$$

With this new estimate, we use again Lemma 3.4 with $\mu_{0}=d-1$ to show that there exists $s_{8}>0$ such that for all $s\geq s_{8}$,

$$\sum_{n=0}^{2}l_{a,n}\,\left(s\right)+r_{a,4}\,\left(s\right)\leq C\,e^{-\frac{s}{2}}\,s^{d-2}$$

and

$$\left|\frac{d}{d\,s}\,\left(g_{a,\beta }\,\left(s\right)\,e^{\frac{s}{2}}\,s^{\left|\bar{\beta }\right|}\right)\right|\leq C\,s^{\left|\bar{\beta }\right|+d-\frac{5}{2}}\;\text{for all }\,\left|\beta \right|=3.$$

This new inequality implies that for all |β|=3 and $s\geq s_{8}$,

• if $\left|\bar{\beta }\right|=0$ or $\left|\bar{\beta }\right|\geq 2$, then $\left|g_{a,\beta }\,\left(s\right)\right|\leq C\,e^{-\frac{s}{2}}\,s^{d-\frac{3}{2}}$,
• if $\left|\bar{\beta }\right|=1$, then we obtain the existence of continuous functions $a\to \lambda_{\beta }\,\left(a\right)$ such that
$$\left|g_{a,\beta }\,\left(s\right)-\frac{e^{-\frac{s}{2}}}{s}\,\lambda_{\beta }\,\left(a\right)\right|\leq C\,e^{-\frac{s}{2}}\,s^{d-\frac{3}{2}}.$$

This concludes the proof of Proposition 2.10. ∎

## References

[1] Bricmont J., Kupiainen A. (1994). Universality in blow-up for nonlinear heat equations. Nonlinearity.

[2] Ebde M. A., Zaag H. (2011). Construction and stability of a blow up solution for a nonlinear heat equation with a gradient term. SeMA J..

[3] Fermanian Kammerer C., Merle F., Zaag H. (2000). Stability of the blow-up profile of nonlinear heat equations from the dynamical system point of view. Math. Ann..

[4] Fermanian Kammerer C., Zaag H. (2000). Boundedness up to blow-up of the difference between two solutions to a semilinear heat equation. Nonlinearity.

[5] Filippas S., Kohn R. V. (1992). Refined asymptotics for the blowup of $u_{t}-\Delta \,u=u^{p}$. Comm. Pure Appl. Math..

[6] Filippas S., Liu W. X. (1993). On the blowup of multidimensional semilinear heat equations. Ann. Inst. H. Poincaré Anal. Non Linéaire.

[7] Herrero M. A., Velázquez J. J. L. (1992). Comportement générique au voisinage d’un point d’explosion pour des solutions d’équations paraboliques unidimensionnelles. C. R. Acad. Sci. Paris Sér. I Math..

[8] Herrero M. A., Velázquez J. J. L. (1992). Generic behaviour of one-dimensional blow up patterns. Ann. Sc. Norm. Super. Pisa Cl. Sci. (4).

[9] Herrero M. A., Velázquez J. J. L. (1993). Blow-up behaviour of one-dimensional semilinear parabolic equations. Ann. Inst. H. Poincaré Anal. Non Linéaire.

[10] Merle F., Zaag H. (1997). Stability of the blow-up profile for equations of the type $u_{t}=\Delta \,u+{\left|u\right|}^{p-1}\,u$. Duke Math. J..

[11] Merle F., Zaag H. (1998). Refined uniform estimates at blow-up and applications for nonlinear heat equations. Geom. Funct. Anal..

[12] Merle F., Zaag H. (2000). A Liouville theorem for vector-valued nonlinear heat equations and applications. Math. Ann..

[13] Nguyen V. T., Zaag H. (2014). Construction of a stable blow-up solution for a class of strongly perturbed semilinear heat equations. http://.

[14] Nguyen V. T., Zaag H. (2015). Finite degrees of freedom for the refined blow-up profile for a semilinear heat equation. http://.

[15] Velázquez J. J. L. (1992). Higher-dimensional blow up for semilinear parabolic equations. Comm. Partial Differential Equations.

[16] Velázquez J. J. L. (1993). Estimates on the (n-1)-dimensional Hausdorff measure of the blow-up set for a semilinear heat equation. Indiana Univ. Math. J..

[17] Zaag H. (2002). One-dimensional behavior of singular *N*-dimensional solutions of semilinear heat equations. Comm. Math. Phys..

[18] Zaag H. (2002). On the regularity of the blow-up set for semilinear heat equations. Ann. Inst. H. Poincaré Anal. Non Linéaire.

[19] Zaag H. (2002). Regularity of the blow-up set and singular behavior for semilinear heat equations. Proceedings of the 3rd International Palestinian Conference on mathematics and Mathematics Education.

[20] Zaag H. (2006). Determination of the curvature of the blow-up set and refined singular behavior for a semilinear heat equation. Duke Math. J..

